# Mechanotransduction Mediated by PDLIM5: The Critical Role of Serpin E2/Integrin β3‐Cytoskeleton‐Nucleoskeleton Axis in Mechanical Osteogenic Programming

**DOI:** 10.1111/cpr.70067

**Published:** 2025-06-03

**Authors:** Yuchao Yang, Shutong Wu, Yining Wang, Jiajun Tang, Jiaxuan Liu, Jinyang Wang, Yunfeng Li, Asmat Ullah Khan, Muhammad Akram Khan, Wenqing Liu, Jinhui Zhu, Konghe Hu, Jingxing Dai, Jun Ouyang

**Affiliations:** ^1^ Yue Bei People's Hospital Postdoctoral Innovation Practice Base Southern Medical University Guangzhou China; ^2^ Guangdong Provincial Key Laboratory of Medical Biomechanics & National Experimental Education Demonstration Center for Basic Medical Sciences (Southern Medical University) & National Key Discipline of Human Anatomy, School of Basic Medical Sciences Southern Medical University Guangzhou China; ^3^ Department of Orthopedics Yue Bei People's Hospital Shaoguan China; ^4^ Xinjin Branch of Chengdu Municipal Public Security Bureau Chengdu China; ^5^ Department of Orthopedics The Third Affiliated Hospital of Southern Medical University Guangzhou China; ^6^ Department of Pathology, Guangdong Provincial People's Hospital Guangdong Academy of Medical Sciences Guangzhou China; ^7^ Hebei University of Chinese Medicine Luquan District, Shijiazhuang City China; ^8^ Department of Veterinary Pathology, Faculty of Veterinary and Animal Sciences PMAS Arid Agriculture University Rawalpindi Pakistan; ^9^ Department of Spine Surgery, Guangdong Provincial People's Hospital Guangdong Academy of Medical Sciences Guangzhou China

**Keywords:** extracellular matrix, human adipose‐derived stem cells, osteogenesis, PDZ and LIM domain 5, serine protease inhibitor E2, tensile stress

## Abstract

Despite the regenerative and self‐repair capabilities of bone tissues, significant bone loss can result in substantial bone defects. This study was aimed at investigating the role and underlying mechanisms of the mechanosensitive protein PDZ and LIM Domain 5 (PDLIM5) in the osteogenic differentiation of human adipose‐derived stem cells (hASCs) under cyclic tensile stress conditions relevant to bone tissue repair. Utilising proteomics and single‐cell RNA sequencing, we identified PDLIM5 and serpin E2 as key genes associated with the osteogenic differentiation of stem cells. To evaluate the expression levels of these genes and related proteins, we utilised western blotting, immunofluorescence and alkaline phosphatase (ALP) staining. Furthermore, lentiviral transfection, Cell Counting Kit‐8 (CCK‐8) assays, transwell migration assays, wound healing assays and protein–protein interaction analyses were conducted to evaluate changes in osteogenic differentiation under both chemical and physical stimuli, as well as to explore the relationship between serine protease inhibitor E2 (serpin E2) and its downstream effector, PDLIM5. The interactions among serpin E2, integrin β3 and PDLIM5 were confirmed through Haematoxylin and Eosin (H&E) staining, immunohistochemistry and immunofluorescence staining of bone tissues and primary adipose‐derived stem cells isolated from integrin β3 knockout mice. Our findings indicate that PDLIM5 modulates the osteogenic differentiation of hASCs via a signalling pathway involving serpin E2, integrin β3 and lamin A.

## Graphical abstract

Large segmental bone defects can result in non‐union of fractures and present a formidable challenge in clinical management. This study aims to investigate the role of mechanical stimulation in regulating osteogenic differentiation of stem cells via the mechanosensitive protein PDLIM5, thereby promoting bone regeneration and repair. Our findings may provide a novel therapeutic target for the clinical treatment of bone defects.

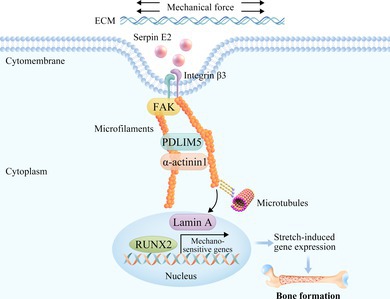

## 1 Introduction

Bone exhibits a certain regenerative capacity and can repair itself to some extent. However, once the critical threshold is exceeded, this ability diminishes, leading to non‐union in approximately 5%–10% of fractures [1, 2]. Therefore, addressing critical bone defects remains a significant global medical challenge. Globally, approximately 4 million people require bone transplantation or bone replacement surgery annually due to large bone defects [3]. However, advancements in bone tissue engineering offer a new therapeutic strategy for repairing bone defects [4]. The ‘diamond concept’, encompassing the mechanical environment, bone‐conductive scaffolds, growth factors and seed cells, emphasises the multifaceted requirements for successful bone regeneration [5]. Nonetheless, the underlying mechanisms through which mechanical signals regulate stem cell osteogenesis remain incompletely understood.

Mechanical factors are associated with almost every stage of development and mechanical stimuli of different types, magnitudes, modes and durations play important roles in cell development and behaviour and enable the mechanical regulation of stem cell differentiation [6]. Mesenchymal stem cells (MSCs), particularly adipose‐derived mesenchymal stem cells (ASCs), exhibit considerable potential for bone regeneration owing to their accessibility and potent osteogenic capacity [7, 8]. Mechanical stimulation, such as cyclic tension, has been demonstrated to enhance osteogenic differentiation; however, the mechanotransduction pathways involved remain incompletely characterised.

The cytoskeleton is essential for mechanical signal transduction [9]. The PDZ‐LIM protein family, including PDLIM1, PDLIM2 and PDLIM5, has been implicated in cytoskeletal organisation and signal transduction. Specifically, overexpression of PDLIM1 inhibits the osteogenic activity of MC3T3‐E1 cells. In vivo studies have also demonstrated that adenovirus‐mediated knockdown of PDLIM1 expression in mice accelerates osteogenesis and fracture healing [10], while PDLIM2 modulates inflammatory responses and cell differentiation via NF‐κB and STAT signalling pathways [11, 12]. However, their roles in osteogenesis remain largely unexplored. In contrast, PDLIM5 exhibits distinct mechanosensitive properties. Our previous work identified PDLIM5 as a mechanosensitive protein that acts as a key mediator linking cytoskeletal dynamics (via α‐actinin1 binding) to nuclear signalling pathways [13]. However, the downstream mechanisms by which mechanosignalling regulates osteogenic genes, such as RUNX2, remain incompletely characterised. Serpin E2, a secreted extracellular matrix (ECM) protein, is crucial for various physiological and pathological processes [14] and has been validated as an upstream interactor of PDLIM5 through bioinformatics and experimental validation. Notably, integrin β3, a well‐recognised mechanical sensor [15], forms a functional axis with serpin E2 and PDLIM5. This study focuses on the serpin E2/integrin β3‐PDLIM5‐nuclear signalling axis, addressing a critical gap in understanding how mechanical signals are decoded into osteogenic signals. While PDLIM1 and PDLIM2 may contribute to wider cytoskeletal regulation, PDLIM5's unique integration of mechanical and transcriptional responses positions it as a pivotal target for bone defect repair. The findings from this study will provide valuable insights and advance targeted strategies for bone defect repair and regeneration.

## 2 Results

### 2.1 Osteogenic Differentiation Promoted by Chemical and Mechanical Stimulation

**FIGURE 1:**
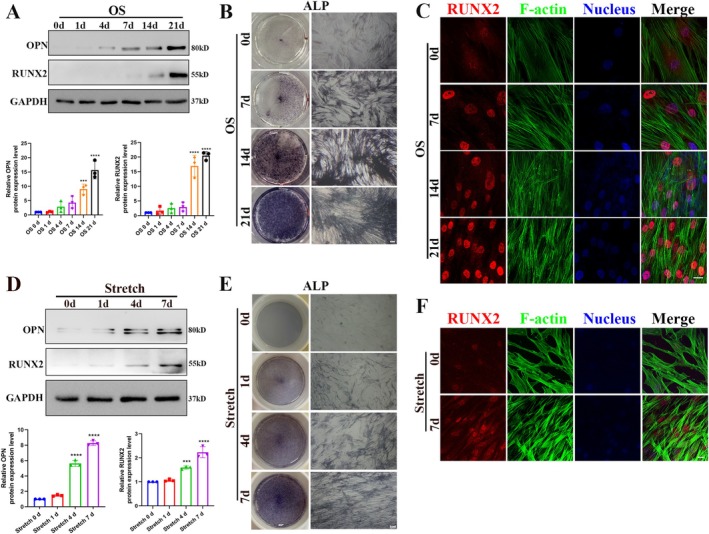
Osteogenic differentiation promoted by chemical and mechanical stimulation. (A) Western blotting analysis shows that the expression levels of runt‐related transcription factor 2 (RUNX2) and osteopontin (OPN) increase in hASCs following chemical‐induced osteogenic differentiation. *p < 0.05, **p < 0.01, ***p < 0.001, ****p < 0.0001, *n* = 3. (B) ALP staining assay results reveal a progressive increase in ALP activity during the osteogenic differentiation of hASCs. Scale bar = 200 μm. (C) Immunofluorescence staining confirms an increase in RUNX2 expression during the osteogenic differentiation of hASCs. Scale bar = 20 μm. (D) Western blotting analysis shows that the expression levels of RUNX2 and OPN increased in hASCs following mechanical stimulation. (E) ALP staining assay results reveal a progressive increase in ALP activity under mechanical stimulation. Scale bar = 200 μm. (F) Immunofluorescence staining confirms an increase in RUNX2 expression during the mechanical stimulation of hASCs. Scale bar = 20 μm.

To ensure the stability of the subsequent experimental process and results, western blotting analysis, ALP staining and immunofluorescence staining were employed to evaluate the osteogenic differentiation ability of hASCs (Figure 1A–C). The cells were cultured in osteogenic medium for 7, 14 and 21 days, and the expression levels of osteoblast‐related markers (OPN and RUNX2) in hASCs were analysed using western blotting (Figure 1A). ALP staining was used to visualise the formation of alkaline phosphatase in hASCs (Figure 1B), and the expression of RUNX2 in the nucleus was detected using immunofluorescence (Figure 1C). The results showed that the expression of osteogenic markers progressively increased with prolonged induction time, indicating the significant osteogenic differentiation potential of hASCs utilised in this study.

Subsequently, hASCs were then cultured on FlexCells 6‐well plates and subjected to 10% cyclic tensile stress (2 h/day) for 7 consecutive days. Western blot analysis showed that osteogenic differentiation markers OPN and RUNX2 gradually increased with the duration of mechanical stimulation and were significantly increased on day 7 compared to days 0 and 1 (Figure 1D). ALP staining further confirmed a gradual increase in alkaline phosphatase expression under mechanical stimulation (Figure 1E). Immunofluorescence staining showed that RUNX2 gradually translocated into the nucleus under cyclic tensile strain, suggesting that mechanical stimulation effectively activates osteogenesis in hASCs (Figure 1F). Collectively, these results suggest that cyclic tensile strain promotes osteogenesis in hASCs.

### 2.2 Effect of PDLIM5 on Osteogenic Differentiation of Stem Cells

**FIGURE 2:**
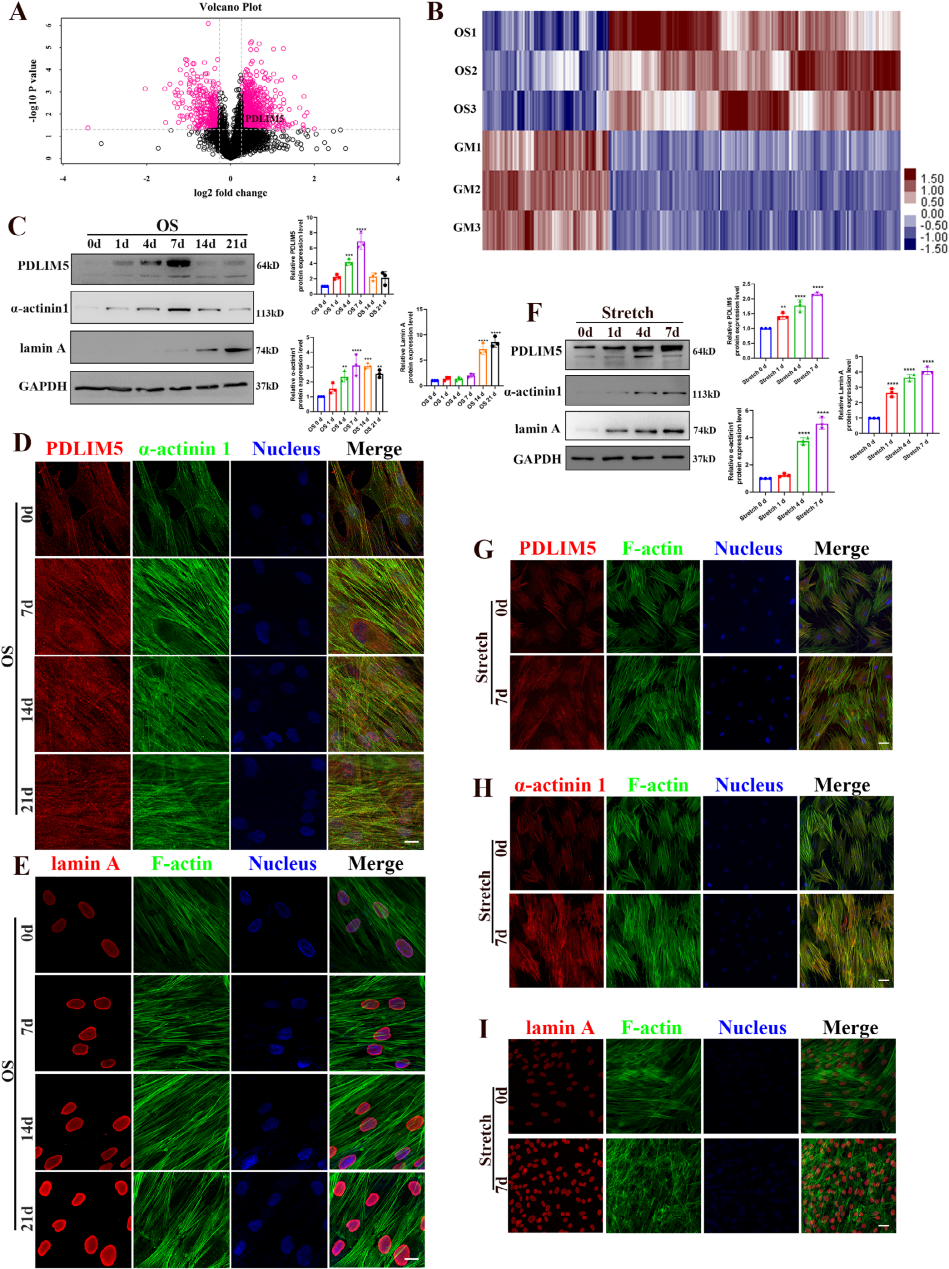
Effect of PDLIM5 on osteogenic differentiation of stem cells. (A) The volcano plot shows the gene information between the positive and negative osteogenic‐related genes. (B) The heat map shows the 998 differentially expressed genes. The pink colour indicates upregulated genes, and the blue indicates downregulated genes. Group GM: Cells treated with growth medium. Group OS: Cells treated with osteogenic differentiation medium. *p* < 0.05, logFC > 1.2. (C) Western blotting analysis shows the expression levels of PDLIM5, α‐actinin 1 and lamin A in hASCs following chemical‐induced osteogenic differentiation. (D, E) Immunofluorescence staining confirms an increase in the expression of PDLIM5, α‐actinin1 and lamin A during osteogenic differentiation of hASCs. Scale bar = 20 μm. (F) Western blotting analysis shows the expression levels of PDLIM5, α‐actinin 1 and lamin A protein increased in hASCs following mechanical stimulation. (G–I) Immunofluorescence staining confirms an increase in the expression of PDLIM5, α‐actinin1 and lamin A during the mechanical stimulation of hASCs. Scale bar = 50 μm.

Comparative proteomics was employed to analyse differential protein changes before and after osteogenic differentiation of hASCs (GM: undifferentiated, OS: osteogenic differentiation for 2 weeks). After 2 weeks of osteogenic differentiation, the expression of PDLIM5 in the OS group was 1.523 times that in the GM group (Figure 2A,B, Figure S1). To investigate the potential roles of PDLIM5 and its related cytoskeleton and nuclear skeleton proteins in the osteogenic differentiation of hASCs, the expressions of PDLIM5, α‐actinin 1 and lamin A during chemically induced osteogenic differentiation were analysed using western blot and immunofluorescence staining (Figure 2C–E). The results revealed that the expression of PDLIM5 and α‐actinin 1 exhibited a synchronised trend, peaking at day 7 and subsequently slightly decreasing (Figure 2C). Immunofluorescence confirmed that both PDLIM5 and α‐actinin 1 were co‐located in stress fibres (Figure 2D), while the nuclear membrane protein lamin A bridged the cytoskeleton and the nuclear skeleton, with its expression significantly upregulated in the osteogenic medium over time (Figure 2E).

To further verify the effects of these proteins on hASCs promoting osteogenesis under a mechanically stimulated environment, hASCs were cultured on a FlexCells 6‐well culture plate for 7 days and subjected to cyclic tensile stress 2 h daily. The results demonstrated the sensitivity of PDLIM5 to mechanical stimulation, with synergistic α‐actinin 1 reaching its peak on the 7th day of the mechanical stimulation (Figure 2F–H). Meanwhile, lamin A played a role in receiving and transmitting mechanical signals during mechanically stimulated osteogenic differentiation of hASCs, with its expression in the nuclear membrane gradually increasing over time (Figure 2I).

### 2.3 Downregulation of PDLIM5 Impairs Osteogenic Differentiation Induced by Chemical and Mechanical Stimulation

**FIGURE 3:**
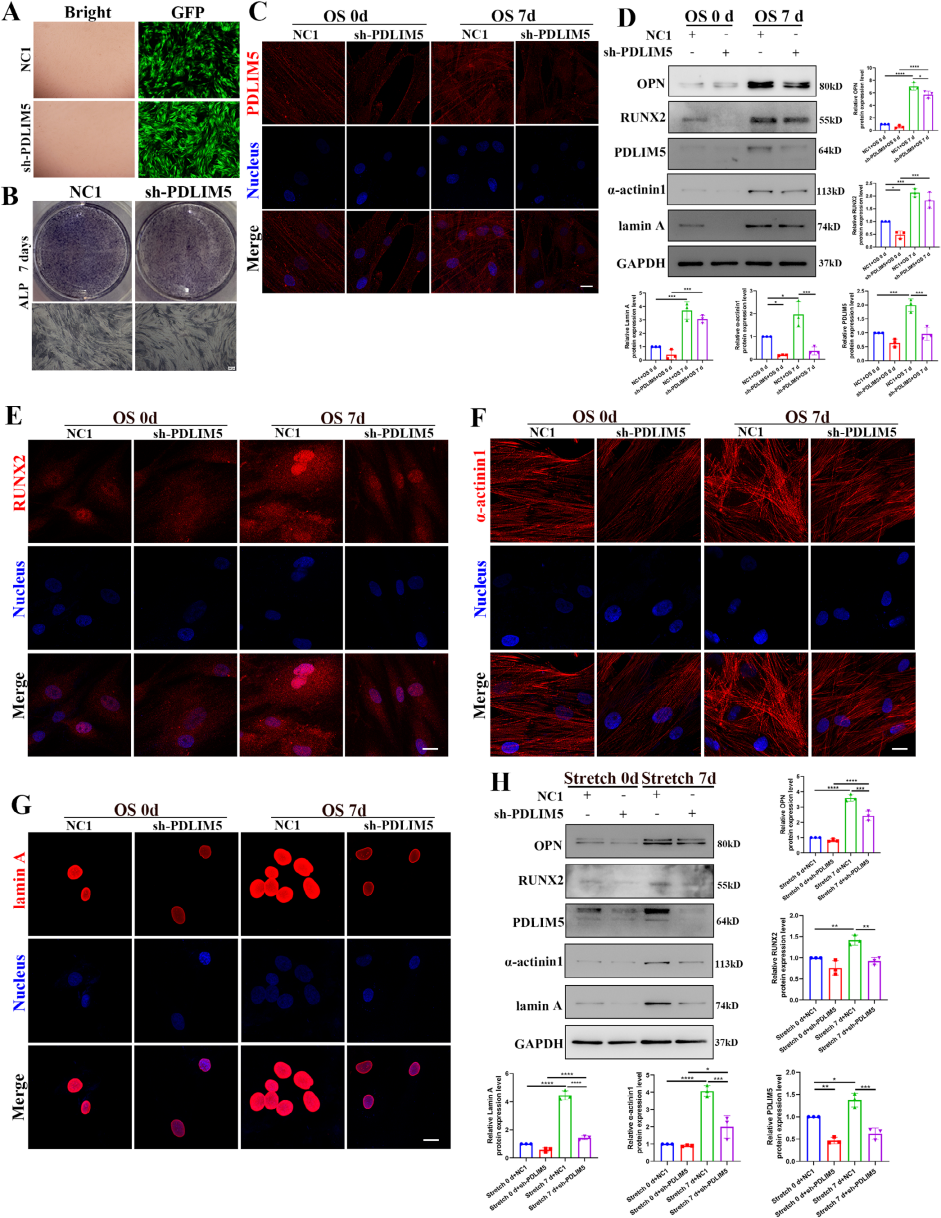
Downregulation of PDLIM5 impairs osteogenic differentiation induced by chemical and mechanical stimulation. (A) Transfection efficiency of the negative control transfected (NC1 group) and PDLIM5 knockdown transfected (shPDLIM5 group) in hASCs. The transfection efficiency was evaluated based on the GFP fluorescence intensity. (B) ALP staining intensity is significantly reduced in shPDLIM5 group compared with the NC1 group when subjected to osteogenic differentiation. (C) Immunofluorescence staining confirms significantly reduced PDLIM5 expression levels in shPDLIM5 group compared with NC1 group. Scale bar = 20 μm. (D) Western blot analysis demonstrates the expression levels of OPN, RUNX2, PDLIM5, α‐actinin1 and lamin A in the NC1 group and shPDLIM5 group. *p < 0.05, **p < 0.01, ***p < 0.001, ****p < 0.0001, *n* = 3. (E–G) Immunofluorescence staining confirms that lentivirus‐mediated PDLIM5 knockdown decreased the expression levels of RUNX2, α‐actinin1 and lamin A in hASCs following chemical‐induced osteogenic differentiation. Scale bar = 20 μm. (H) Western blotting analysis demonstrates the expression levels of OPN, RUNX2, PDLIM5, α‐actinin1 and lamin A in NC1 group and shPDLIM5 group under cyclic stretch loading.

Lentiviral vector‐transfected cells were utilised to reduce the expression of PDLIM5 during the osteogenic differentiation of hASCs, thereby exploring the mechanosensitive function of PDLIM5. Based on the preliminary experiments, an MOI of 40 was selected as the optimal multiplicity of infection under the premise of minimising the number of viruses and ensuring the transfection efficiency of 80%–90% (Figure 3A). The knockdown efficiency of PDLIM5 in hASCs was further validated using ALP staining, immunofluorescence and western blotting analysis (Figure 3B–D).

The effect of PDLIM5 knockdown on hASCs behaviour was assessed through relevant experiments. CCK‐8 assay revealed that PDLIM5 knockdown significantly reduced the proliferation rate of hASCs (Figure S2A). Similarly, migration and wound healing tests demonstrated a significant decline in cell motility (Figure S2B,C). These findings suggest that PDLIM5 is involved in regulating the growth cycle and daily activities of hASCs, playing an important role in modulating their cellular behaviour.

In PDLIM5‐knockdown hASCs, the expression intensity of the early osteogenic marker ALP decreased on day 7 of osteogenic differentiation, as indicated by ALP staining (Figure 3B). Additionally, the expression levels of osteoblastic markers OPN and RUNX2 were significantly lower compared to the non‐knockdown group (Figure 3D,E).

Downregulation of PDLIM5 also affected α‐actinin 1, which was co‐localised to stress fibres, exhibiting a synchronous downward trend (Figure 3D,F). The expression of lamin A, a downstream nuclear membrane protein, was reduced due to the downregulation of PDLIM5, thereby impairing the osteogenic differentiation capacity of hASCs (Figure 3G).

The effect of PDLIM5 knockdown on promoting osteogenic differentiation of hASCs under mechanical stimulation was evaluated using western blot analysis. The results indicated that under cyclic tensile stress, the expressions of osteogenic differentiation markers OPN and RUNX2 decreased due to PDLIM5 downregulation. Additionally, the expression of α‐actinin 1, closely associated with the localisation and function of PDLIM5, was also diminished (Figure 3H). Notably, the expression of downstream lamin A was significantly reduced on the 7th day of cyclic stress stretching in the PDLIM5‐knockdown group compared with the non‐knockdown control group (Figure 3H). These findings indicate that PDLIM5 downregulation impairs the expression and co‐localisation of α‐actinin 1 bound to actin stress fibres under mechanical stimulation, thereby weakening the progressive transmission of mechanical signals generated by the cytoskeleton in response to mechanical cues. The reduction in lamin A expression further verified the critical role of PDLIM5 knockdown in disrupting the downstream mechanical signal transmission. In summary, our findings indicated that PDLIM5, as a cytoskeleton‐related protein, plays a pivotal role in mediating mechanical signal transmission from upstream α‐actinin 1 to the nucleus, where they are integrated into corresponding intracellular chemical signals.

### 2.4 Bioinformatics Analysis of PDLIM5 Upstream Protein and Differential Gene Expression in Osteogenic Differentiation

**FIGURE 4:**
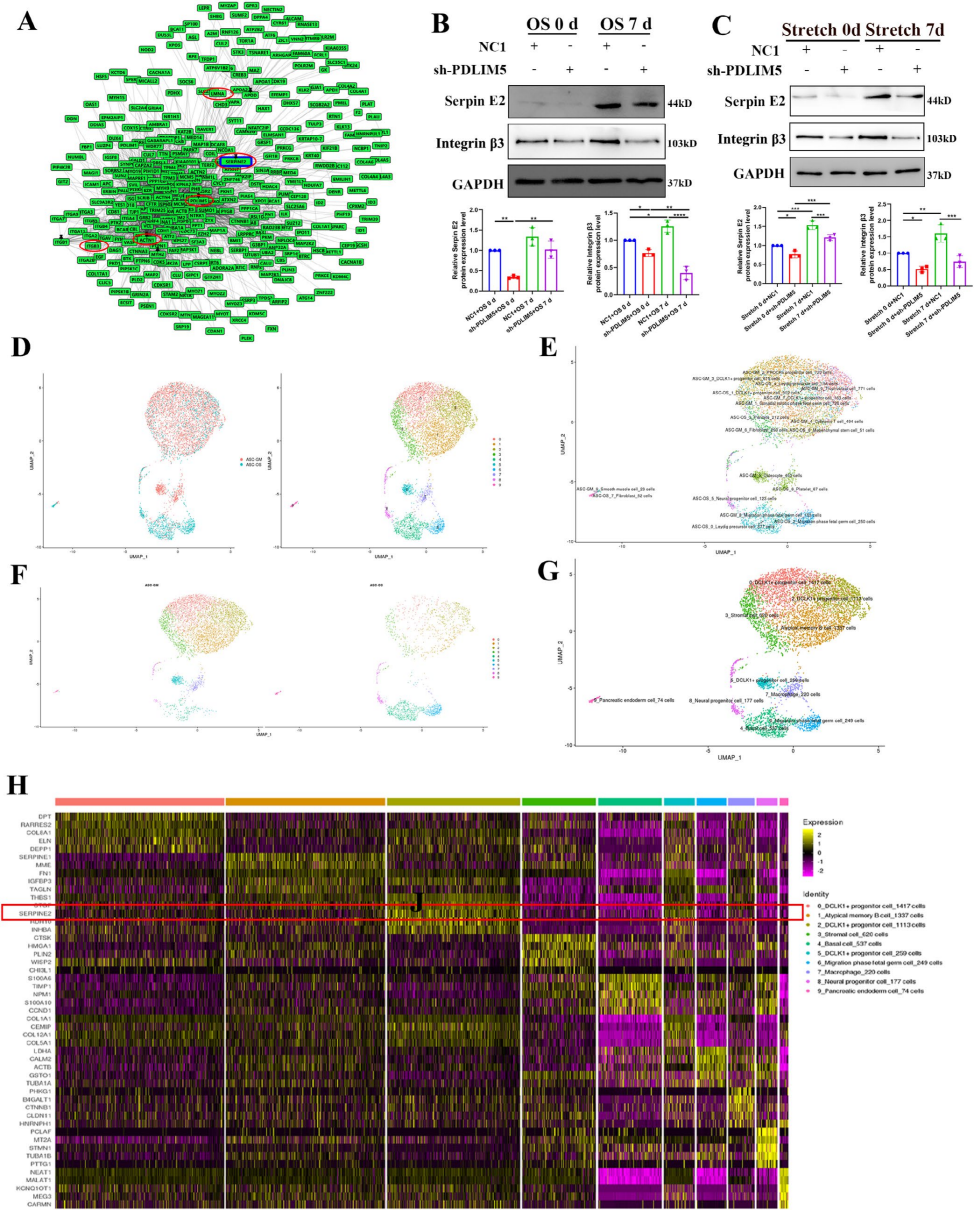
Bioinformatics analysis of PDLIM5 upstream protein and differential gene expression in osteogenic differentiation. (A) Protein–Protein Interaction network of serpin E2 interacting with integrin β3‐PDLIM5‐lamin A. (B) Western blot analysis shows the expression levels of serpin E2 and integrin β3 in NC1 group and shPDLIM5 group induced by chemical. *p < 0.05, **p < 0.01, ***p < 0.001, ****p < 0.0001, *n* = 3. (C) Western blotting analysis shows the expression levels of serpin E2 and integrin β3 in NC1 group and shPDLIM5 group under cyclic stretch loading. (D) UMAP clustering results show the top 30 principal components and the distribution of hASCs‐GM and hASCs‐OS samples. Uniform manifold approximation and projection (UMAP) plots show the origin of the hASCs‐GM cells (*n* = 3687) and the hASCs‐OS cells (*n* = 5511) and the 10 cell clusters (0–9). (E) The distribution of subpopulations of cells in the two samples (hASCs‐GM and hASCs‐OS) based on the integrated data from the UMAP clustering results. (F) UMAP clustering of the subgroup of cells based on the integrated data from the two samples. (G) Distribution of the number of cells in each subgroup based on the integrated data from the UMAP clustering results. (H) Heatmap shows the top five tag genes in different subgroups. Each row represents a gene; each column represents a single cell; colour depth represents the intensity of gene expression in the samples from the hASCs‐GM and hASCs‐OS groups.

Bioinformatics analysis of genetic and protein–protein interaction data between the human immunodeficiency virus (HIV) and the human host based on GPS‐Prot (http://www.gpsprot.org) suggested that PDLIM5 had upstream and downstream interactions with serpin E2 and integrin β3. This interaction is significant for exploring relevant key proteins on the mechanical signalling pathway (Figure 4A). To preliminarily verify the relationship between PDLIM5 and serpin E2, hASCs were cultured under osteogenic medium and cyclic stress stretching conditions for 7 days. Our findings demonstrated that downregulation of PDLIM5 expression would affect the expression of serpin E2 and integrin β3 in hASCs under both conditions (Figure 4B,C).

To further investigate the role of serpin E2 in the osteogenic differentiation of hASCs and its relationship with PDLIM5, we assessed changes in serpin E2 expression in osteogenic differentiation. We also examined the changes of different cell populations and hASCs during osteogenic differentiation using single‐cell RNA sequencing. We obtained 3687 cells from the undifferentiated cells (hASCs‐GM) group after 1 week in the growth medium and 5511 cells from the induced osteogenesis cells (hASCs‐OS) group after 1 week of growth in the osteogenic induction medium. After cell and gene filtering, the high‐throughput single‐cell transcriptome was quantified using the barcode sequence markers in the sequencing data from each cell, and the unique molecular identifier (UMI) markers of different mRNA molecules in each cell were evaluated. The integrated sample cells could be divided into 10 subgroups (0–9 cluster), and most of the hASCs‐GM cells were well separated from the hASCs‐OS cells, and only marginal overlap was observed between the hASCs‐GM and hASCs‐OS (Figure 4D,E). We also plotted the distribution of subgroups of the two samples in the integrated data clustering (Figure 4E) and the clustering of each subgroup of the integrated data in the two samples (Figure 1F,G).

Subgroup distribution and clustering on UMAP plots indicated a homogeneous gene expression pattern within clusters. As only a few hASCs had begun to differentiate into osteoblasts during the first week of osteogenic differentiation, hASCs‐GM cells were dominant in the 0–3, 5, 7 cluster, while the cells in cluster 4, 6, 8, 9 were from the hASCs‐OS group. The hASCs in cluster 2 were characterised by the expression of fibronectin 1 (FN1), serine protease inhibitor 2 (SERPINE2), insulin‐like growth factor binding‐protein 3 (IGFBP3), thrombospondin 1 (THBS1) and Cellular Communication Network Factor 2 (CTGF; Figure 1H).

The top two tag genes (ordered by ratio) in different subgroups were selected from the expression profiles and plotted (Figure S3). The top five tag genes across all the subgroups were IGFBP3, SERPINE1, SERPINE2, FN1 and MALAT1 (Figure S3). The top two differentially expressed genes from each subgroup were selected and visualised in all the subgroups of cell clusters using the expression distribution map (Figure S4) and the violin plot (Figure S5). Overall, we identified 18 genes, including SERPINE2 as shown in Figure S3.

### 2.5 Effects of Serpin E2 on PDLIM5 and Integrin β3 During Osteogenic Differentiation

**FIGURE 5:**
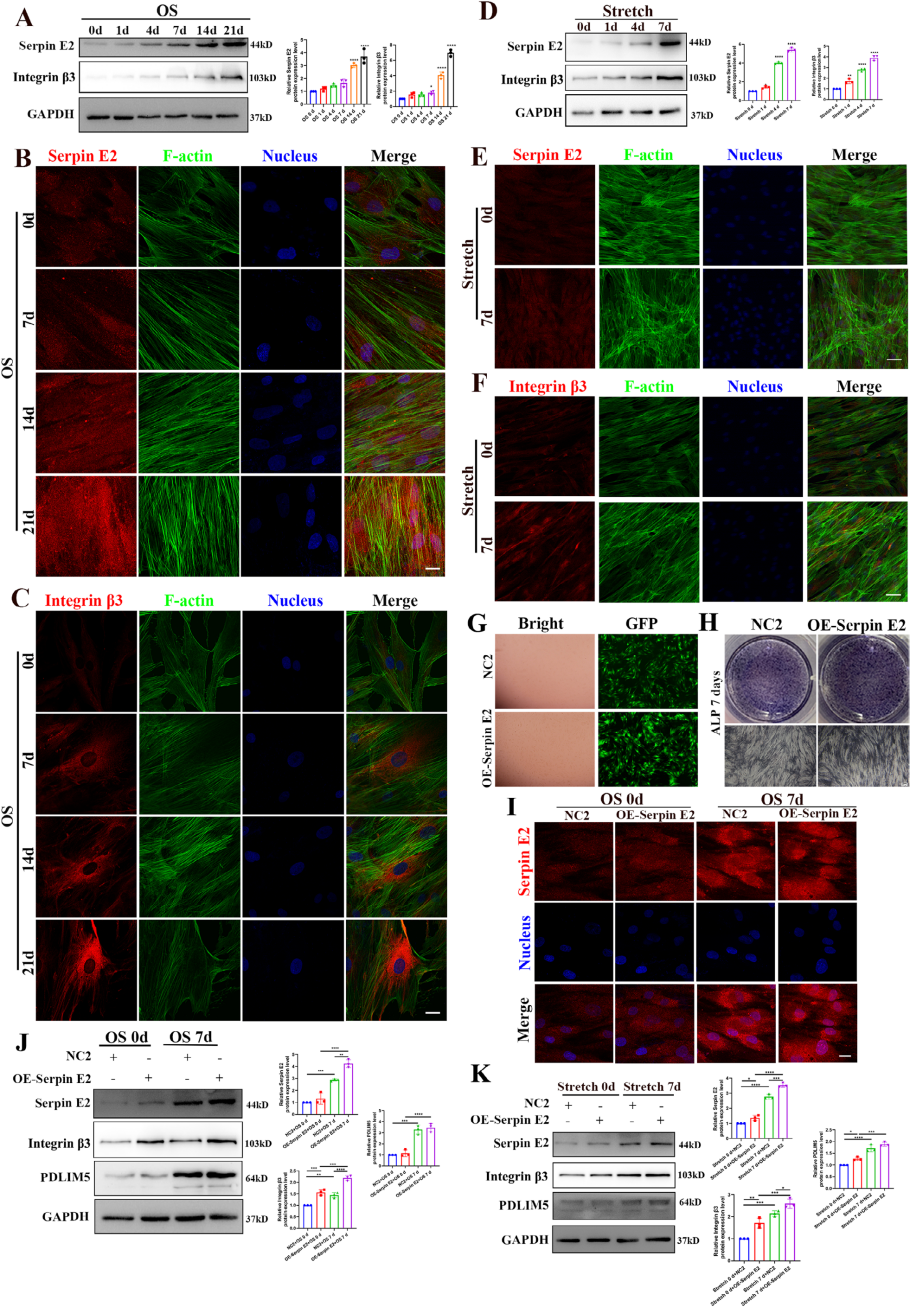
Effects of serpin E2 on PDLIM5 and integrin β3 during osteogenic differentiation. (A) Western blotting results show the expression levels of serpin E2 and integrin β3 in the hASCs following chemical‐induced osteogenic differentiation. (B, C) Immunofluorescence staining confirms an increase in serpin E2 and integrin β3 expression during osteogenic differentiation of hASCs. Scale bar = 20 μm. (D) Western blotting results show that the expression levels of serpin E2 and integrin β3 increased in hASCs following mechanical stimulation. (E, F) Immunofluorescence staining confirms an increase in the expression of serpin E2 and integrin β3 during the mechanical stimulation of hASCs. Scale bar = 50 μm. (G) Transfection efficiency of the negative control transfected (NC2 group) and serpin E2‐overexpressing transfected (OE‐Serpin E2 group) in hASCs. The transfection efficiency was evaluated based on the GFP fluorescence intensity. (H) Serpin E2‐overexpressing hASCs exhibit significantly higher ALP activity compared with the NC2 group when subjected to osteogenic differentiation. (I) Immunofluorescence analysis reveals that serpin E2 expression levels were significantly higher in the OE‐Serpin E2 group compared with the NC2 group following osteogenic differentiation. Scale bar = 20 μm. J. Western blotting results show the expression levels of serpin E2, integrin β3 and PDLIM5 in NC2 group and OE‐Serpin E2 group when subjected to osteogenic differentiation. *p < 0.05, **p < 0.01, ***p < 0.001, ****p < 0.0001, *n* = 3. (K) Western blotting analysis shows the expression levels of serpin E2, integrin β3 and PDLIM5 in NC2 group and the OE‐Serpin E2 group under cyclic stretch loading. *p < 0.05, **p < 0.01, ***p < 0.001, ****p < 0.0001.

To further investigate the effects of serpin E2 changes during osteogenic differentiation and its effects on upstream and downstream proteins, western blot analysis revealed that the expression of serpin E2 and integrin β3 gradually increased with the prolonged osteogenic stimulation time under both chemical and mechanical stimulation conditions, respectively (Figure 5A,D). Immunofluorescence analysis also confirmed the continuous upregulation of serpin E2 in the cytoplasm and integrin β3 on the membrane (Figure 5B,C,E,F), preliminarily validating their regulatory roles in the osteogenic differentiation of hASCs.

To further substantiate the role of serpin E2 in osteogenic differentiation of hASCs and its relationship with PDLIM5 and integrin β3, lentivirus transfection was employed to upregulate serpin E2 expression (Figure 5G). Under both stimulation conditions, ALP staining indicated enhanced expression of alkaline phosphatase, an early marker of osteogenesis, following serpin E2 upregulation (Figure 5H), indicating the promotion of osteogenic differentiation upon the overexpression of serpin E2 (Figure 5I). Western blot analysis further demonstrated that the expression of PDLIM5 and integrin β3 was significantly increased under osteogenic stimulation after serpin E2 overexpression (Figure 5J,K), suggesting potential interactions among these three proteins. These findings suggest that serpin E2, PDLIM5 and integrin β3 play critical roles in promoting the osteogenic differentiation of hASCs and affecting bone regeneration in mechanically stimulated environments.

### 2.6 Overexpression of Serpin E2 Affects the Regulation of the Signalling Axis in Osteogenic Differentiation of Stem Cells

**FIGURE 6:**
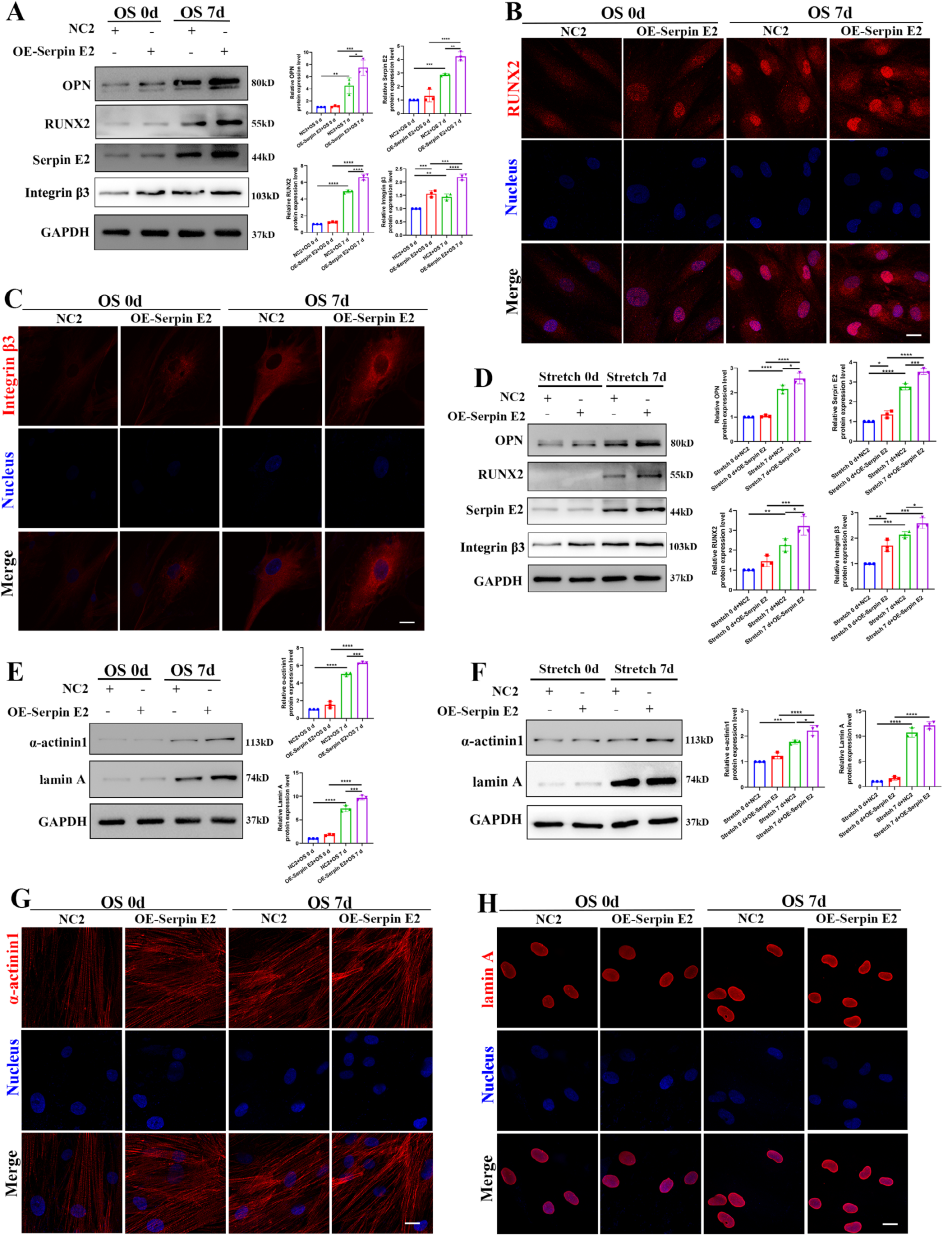
Overexpression of Serpin E2 affects the regulation of the signalling axis on the osteogenic differentiation of stem cells. (A) Western blotting analysis demonstrates the expression levels of OPN, RUNX2, serpin E2 and integrin β3 in NC2 group and OE‐Serpin E2 group during osteogenic differentiation. *p < 0.05, **p < 0.01, ***p < 0.001, ****p < 0.0001, *n* = 3. (B, C) Immunofluorescence analysis confirms that lentivirus‐mediated overexpression of serpin E2 enhances the expression of RUNX2 and integrin β3 in hASCs during osteogenic differentiation. (D) Western blotting analysis reveals the expression levels of OPN, RUNX2, serpin E2 and integrin β3 in the NC2 group and OE‐Serpin E2 group under cyclic stretch loading. (E) Western blotting analysis demonstrates the expression levels of α‐actinin1 and lamin A in the NC2 group and OE‐Serpin E2 group during osteogenic differentiation. (F) Western blotting analysis demonstrates the expression levels of α‐actinin1 and lamin A in the NC2 group and OE‐Serpin E2 group under cyclic stretch loading. (G, H) Immunofluorescence analysis confirms that lentivirus‐mediated overexpression of serpin E2 enhances the expression of α‐actinin1 and lamin A in hASCs during osteogenic differentiation.

The influence of serpin E2 on the mechanical signalling axis during osteogenic differentiation of hASCs was further investigated. Under osteogenic medium conditions, the overexpression of serpin E2 resulted in a significant increase in the expression levels of osteogenic differentiation markers OPN and RUNX2 (Figure 6A). Immunofluorescence analysis revealed significant aggregation of RUNX2 in the nucleus (Figure 6B). Additionally, the expression of integrin β3, a cell membrane signal receptor, was influenced by serpin E2 levels, with its expression also significantly upregulated (Figure 6A), as confirmed by immunofluorescence showing enhanced expression on the cell membrane (Figure 6C). Similarly, in the mechanical stimulation environment, the upregulation of serpin E2 resulted in a more pronounced osteogenic differentiation effect, characterised by increased marker expression and elevated levels of integrin β3 (Figure 6D). These findings suggest that serpin E2 modulates its function to adapt to external environmental changes during osteogenic differentiation.

We also preliminarily investigated whether changes in serpin E2 expression could influence the expression of cytoskeleton‐associated protein α‐actinin 1 and lamin A, a downstream nuclear membrane protein. Consistent with upstream‐related proteins, α‐actinin 1 and lamin A expression exhibited a comparable upward trend in both stimulation environments (Figure 6E–H). These findings collectively suggest that serpin E2, as an upstream regulator, plays a significant role in modulating the PDLIM5‐mediated signalling axis during osteogenic differentiation.

### 2.7 Effect of Integrin β3 Deletion on SerpinE2/PDLIM5 Axis‐Mediated Bone Regeneration In Vivo

**FIGURE 7:**
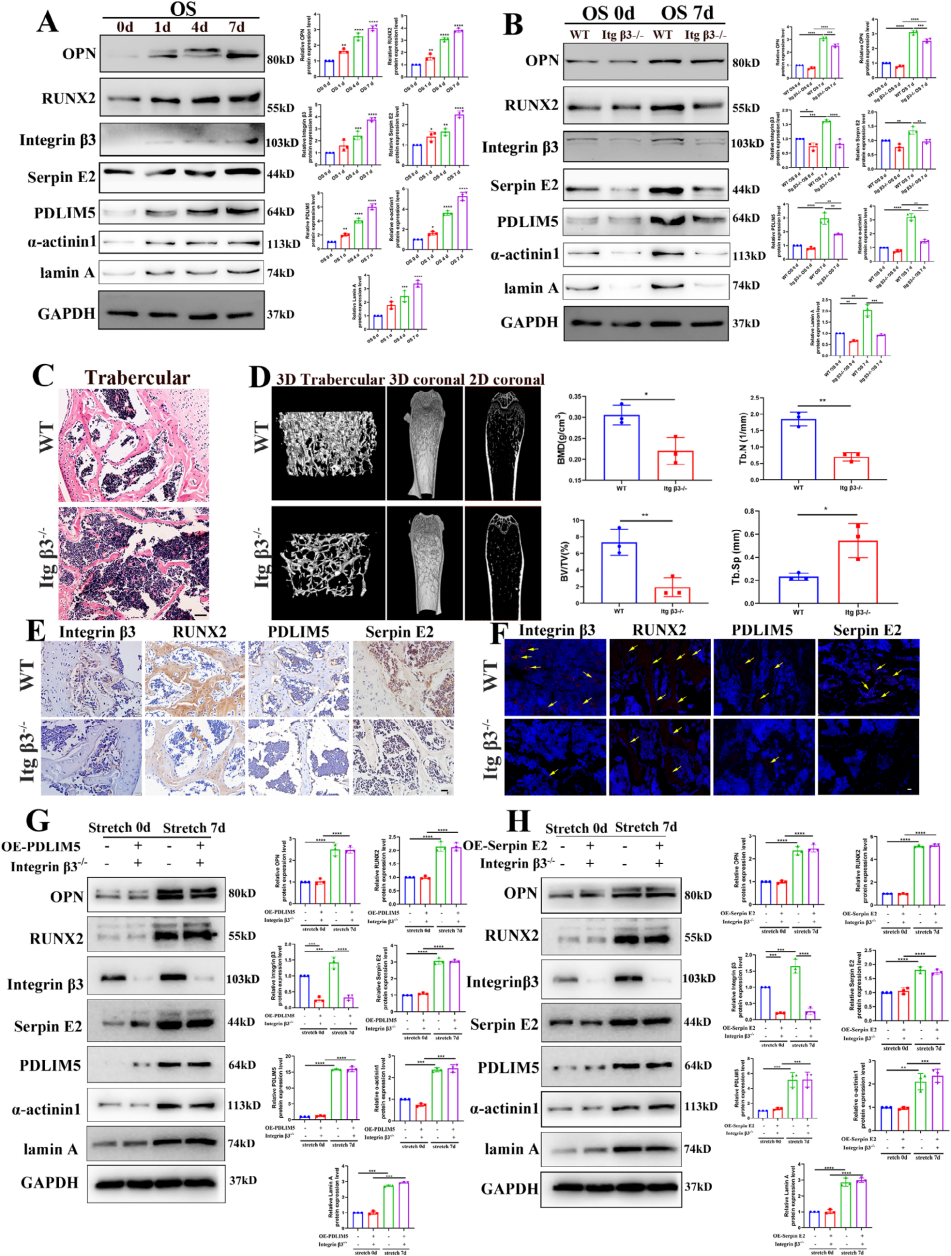
Effect of integrin β3 deletion on SerpinE2/PDLIM5 axis‐mediated bone regeneration in vivo. (A) Western blotting results show that the expression levels of OPN, RUNX2, integrin β3, serpin E2, PDLIM5, α‐actinin1 and lamin A in mouse adipose‐derived stem cells (mASCs) following chemical‐induced osteogenic differentiation. B. Western blot analysis reveals the expression levels of OPN, RUNX2, integrin β3, serpin E2, PDLIM5, α‐actinin1 and lamin A in mASCs isolated from wild‐type (WT) and integrin β3 knockout (KO) mice. (C) H&E staining results indicate that the thickness and continuity of the trabecular bone are significantly reduced in integrin β3 KO mice compared to WT mice. Scale bar = 50 μm. (D) Representative 3D reconstruction, micro‐CT analysis of the entire distal femur in two different groups. Quantitative analysis of bone mineral density (BMD), bone volume/tissue volume (BV/TV), trabecular number (Tb.N) and trabecular thickness (Tb.Th) of cancellous bone. (E) Immunohistochemical staining confirms that the expression of integrin β3, RUNX2, PDLIM5 and serpin E2 is significantly reduced in the bone tissues of integrin β3 KO mice compared with those from WT mice. Scale bar = 20 μm. (F) Tissue immunofluorescence staining analysis confirms the reduced expression of integrin β3, RUNX2, PDLIM5 and serpin E2 in the bone tissues of integrin β3 KO mice compared with those from WT mice. Scale bar = 20 μm. (G) Western blotting analysis demonstrates the expression levels of OPN, RUNX2, integrin β3, serpin E2, PDLIM5, α‐actinin 1 and lamin A in control and PDLIM5‐overexpressing hASCs under cyclic stretch loading. *p < 0.05, **p < 0.01, ***p < 0.001, ****p < 0.0001, *n* = 3. (H) Western blotting analysis demonstrates the expression levels of OPN, RUNX2, integrin β3, serpin E2, PDLIM5, α‐actinin 1 and lamin A in control and serpin E2‐overexpressing hASCs under cyclic stretch loading. *p < 0.05, **p < 0.01, ***p < 0.001, ****p < 0.0001, *n* = 3.

Western blot analysis revealed that the expression levels of serpin E2, integrin β3, PDLIM5, α‐actinin1, lamin A, RUNX2 and OPN proteins increased gradually from day 0 to day 7 in the wild‐type mouse adipose‐derived stem cells (mASCs) after osteogenic induction (Figure 7A). In contrast, mASCs derived from the integrin β3 knockout group exhibited significantly reduced expression levels of serpin E2, PDLIM5, α‐actinin1, lamin A, RUNX2 and OPN compared to those in wild‐type mASCs (Figure 7B).

H&E staining and micro‐CT analysis demonstrated that the bone trabecular density and continuity were significantly decreased in the integrin β3 knockout mice compared with wild‐type mice (Figure 7C,D). Immunohistochemical analysis further demonstrated that the staining intensities of integrin β3, RUNX2, serpin E2 and PDLIM5 in the bone sections were significantly reduced in the integrin β3 knockout mice relative to wild‐type mice (Figure 7E). Immunofluorescence analysis of mouse bone tissue further confirmed that the staining intensity of integrin β3, serpin E2, PDLIM5 and the osteogenic marker RUNX2 in the bone sections of integrin β3 knockout mice was significantly lower in bone sections from integrin β3 knockout mice than in those from wild‐type mice (Figure 7F). These results suggested that reduced osteogenic differentiation of the integrin β3 knockout mASCs is closely associated with reduced levels of serpin E2 and PDLIM5. Furthermore, these results indicate that integrin β3 signalling plays a critical role in stimulating serpin E2 upregulation during osteogenic differentiation.

**FIGURE 8:**
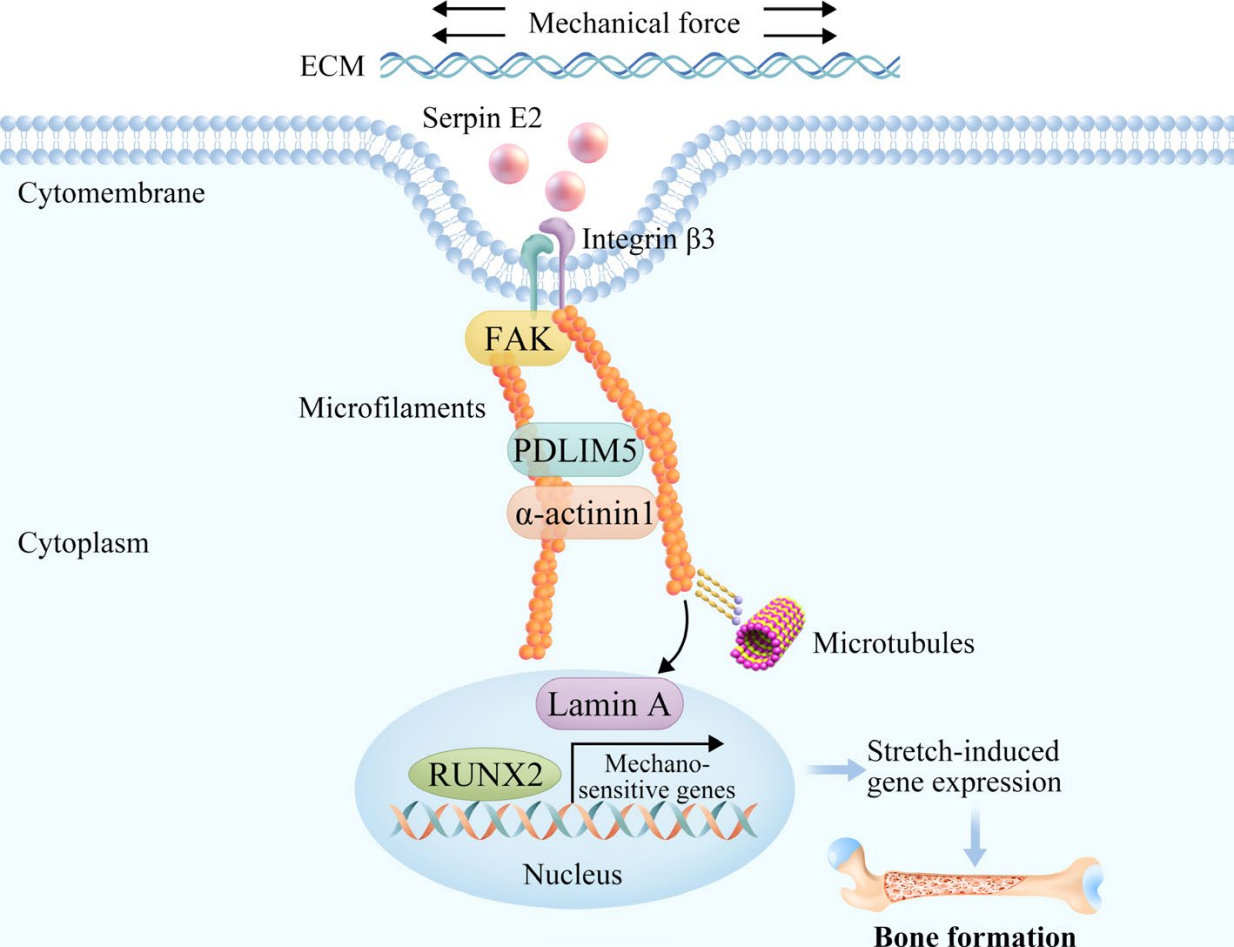
The schematic diagram of the regulatory mechanism illustrates that PDLIM5 mediates mechanical stimulation, thereby promoting osteogenic differentiation of human adipose‐derived stem cells (hASCs) via the serpin E2/integrin β3‐PDLIM5‐lamin A signalling axis.

To further investigate the functional roles of PDLIM5 and serpin E2 in this signalling axis, we conducted a rescue experiment by enhancing the expression of PDLIM5 and serpin E2 in integrin‐β3‐deficient mASCs and applying mechanical cyclic stretching stimulation for 7 days. Western blot analysis showed that the expression levels of osteoblast protein markers OPN and RUNX2 did not exhibit a significant decrease, while integrin β3 remained reduced. Additionally, mechanically related signalling proteins serpin E2, PDLIM5, α‐actinin1 and lamin A did not show significant reductions (Figure 7G,H). These findings suggest that in the absence of integrin β3, overexpression of PDLIM5 and serpin E2 can partially restore the regulation of osteogenic differentiation of stem cells under mechanical stimulation. These results demonstrated that PDLIM5 and serpin E2 play crucial roles in regulating the serpin E2/integrin β3‐cytoskeleton‐nucleoskeleton mechanical signalling axis (Figure 8).

## 3 Discussion

Human adipose‐derived stem cells (hASCs) are extensively utilised as seed cells in tissue engineering owing to their remarkable capacity for multilineage differentiation, thereby providing significant potential for applications in regenerative medicine [16, 17]. Therefore, an in‐depth exploration of the mechanisms underlying the osteogenic differentiation of mesenchymal stem cells (MSCs) is essential for enhancing bone defect repair and addressing associated diseases. Mechanical stimulation represents a pivotal factor that influences the fate determination of MSCs. Through mechanotransduction pathways, such stimuli can directly or indirectly modulate the expression of genes linked to osteogenesis, thereby guiding stem cell development toward specific lineages [18].

In this study, we systematically investigated the effects of osteogenic differentiation in hASCs under both chemical and mechanical stimuli (Figure 1). Proteomic analysis results indicate that PDLIM5 is closely associated with dynamic cytoskeletal remodelling and plays a pivotal role in regulating signal transduction pathways, significantly contributing to the osteogenic differentiation of hASCs (Figure 2A,B, Figure S1). Furthermore, the complex interdependent relationship among PDLIM5, serpin E2 and integrin β3 (Figure 4) underscores the critical roles of these proteins in modulating the functional behaviour of hASCs during osteogenic differentiation. Additionally, animal experiments revealed that integrin β3 deficiency resulted in both systemic structural alterations in mouse models and a significant reduction in trabecular density in specific regions, such as the femur. Concurrently, the downregulation of PDLIM5 expression, along with serpin E2, adversely impacted bone homeostasis in vivo (Figure 7), further substantiating the critical role of this pathway in maintaining systemic bone formation equilibrium.

Furthermore, we utilised CCK‐8 assays, wound healing experiments and transwell migration assays to systematically evaluate the effects of PDLIM5 knockdown or serpin E2 overexpression on the proliferation and migratory capacity of hASCs (Figures S2 and S6). Our findings demonstrate that PDLIM5 knockout significantly diminishes the proliferation and migration capacity of hASCs (Figure S2), consistent with previous studies on the ability of PDLIM5 to suppress metastasis and invasion in tumour cells [19]. This highlights the critical importance of PDLIM5 in maintaining fundamental cellular functions. Such effects may be attributed to the distinct functional roles of its two structural domains. Additionally, research has suggested that PDLIM5 facilitates proximity between AMPK and F‐actin through its PDZ and LIM domains, promoting remodelling of actin filament structures, which subsequently influences cell migration [20].

The mechanical properties of the ECM in various tissues play a crucial role in modulating stem cell differentiation into distinct lineages through signalling pathways involving the cytoskeleton [21]. Alpha‐actinin is essential for maintaining strict alternating crosslinks between actin filaments [22]. The PDZ domain interacts with α‐actinin at the integrin‐localised adhesion junction to regulate the biological activity of the cytoskeleton, while the LIM domain binds to and recruits kinases to the stress fibre [23, 24, 25]. Both α‐actinin 1 and α‐actinin 4 proteins are localised at the leading edge of motile cells, participating in cell adhesion and focal contacts, as well as along actin stress fibres during cellular migration [26]. Specifically, α‐actinin 1 has been implicated in osteogenic differentiation of human skin fibroblasts (HSFs), with the expression levels of both PDLIM5 and α‐actinin 1 increasing over time [27].

In this study, we observed a time‐dependent increase in α‐actinin1 and PDLIM5 protein levels during the 0–7 days of chemical and mechanical induction of hASCs for osteogenic differentiation (Figure 2C,F). Enhanced expression and co‐localisation of α‐actinin1 and PDLIM5 proteins were detected during the chemical induction (Figure 2D), while these proteins co‐localised with microfilament within the cell cytoskeleton during mechanical stimulation (Figure 2G,H). These observations indirectly indicate that both proteins are closely related to stress fibre formation. In our study, PDLIM5 protein expression peaked after 7 days in chemical induction (Figure 2C), whereas PDLIM5 protein increased gradually with the duration of mechanical stimulation, reaching its highest level on day 7 (Figure 2F). This time point differs from the previously reported osteogenic differentiation time point of HSFs [27]. This discrepancy may be attributed to the difference between the two cell types or modes of action rather than experimental errors. These results suggest that PDLIM5 promotes the osteogenic differentiation of hASCs by binding to α‐actinin 1 and regulating microfilament cytoskeleton assembly and function. This finding highlights the necessity for further exploration of the potential signalling pathways between these two proteins, as well as their collaborative role in regulating the fate‐determining processes of MSCs.

Mechanical signalling pathways transmit stimuli from the extracellular environment to the intracellular space, traversing multiple cellular components before reaching the nucleus and triggering gene expression. Throughout these stages, signals must be precisely regulated to ensure their eventual arrival at the nucleus for gene expression activation [28]. However, there remains a significant gap in our current understanding of how external mechanical signals are specifically conveyed to key nuclear proteins, rendering this area an important and challenging field of research. In this context, the cytoskeleton assumes an indispensable role. The cytoskeleton not only regulates vesicular transport and mediates cell signalling but also primarily provides mechanical stability to cells while facilitating the transfer of mechanical signals from the cytoplasm to the nucleus, enabling rapid responses to environmental changes across different types of stimuli [29]. Lamin A serves as a critical link between the cytoskeleton and nuclear scaffold, endowing the nucleus with the mechanical stability necessary for adaptation to fluctuating mechanical microenvironments. It also plays an essential role in transducing mechanical signals into biological responses that influence cell survival, growth, differentiation and disease progression [30, 31]. Consequently, elucidating the function of lamin A within the nucleus is pivotal for uncovering underlying mechanisms associated with human diseases. During mechanically stimulated osteoblast differentiation, MKL1 and β‐catenin translocation is dependent on lamin A/C [32]. In stem cells undergoing osteogenic differentiation induced by cyclic stress stretching, both lamin A and PDLIM5 expressions have been observed to increase concurrently (Figure 2G,I). This observation suggests that these two proteins may have a synergistic relationship and are involved in regulating the bone formation process. Further validation analyses indicate that downregulation of PDLIM5 expression corresponded with a decrease in lamin A levels (Figure 3H), indicating that PDLIM5 may regulate lamin A to participate in the nuclear transduction effects induced by mechanical stimulation. Therefore, further exploration of these mechanisms may clarify how to improve or treat related diseases by manipulating these key factors.

Serpin E2 is an extracellular matrix protein that interacts with multiple proteins within the ECM and is related to various bone and cartilage diseases. For example, serpin E2 prevents the degradation of articular cartilage and protects joints by inhibiting inflammation‐induced ECM degradation through the induction of matrix metalloproteinases overexpression [33]. Additionally, serpin E2 has been identified as a key upregulated gene that alleviates steroid‐induced femoral head necrosis by enhancing cell survival, offering a novel therapeutic approach for related diseases [34]. Serpin E2 also plays an important role in bone formation and bone repair. Preliminary single‐cell transcriptome sequencing analysis (Figure 4D–H, Figures S3–S5) revealed that the expression of serpin E2 significantly increased during the osteogenic differentiation of hASCs, suggesting that its protein may play a regulatory role in the transformation of stem cells into osteoblasts [35]. We detected a steady time‐dependent increase in serpin E2 levels during osteogenic‐induced hASCs from days 0 to 21 (Figure 5A,B), along with a gradual increase under mechanical stimulation conditions (Figure 5D,E). These observations indicate that serpin E2 positively regulates its role in the osteogenic differentiation of stem cells. Given its role as an important anti‐apoptotic factor, it may influence stem cells fate by modulating specific structural or functional regions within the ECM. Consequently, we hypothesise that serpin E2 knockdown could lead to increased ECM degradation levels, resulting in more stem cells undergoing apoptosis [36]. Subsequently, we systematically analysed the effects of serpin E2 overexpression on various biological behaviours of hASCs, including proliferation, migration and osteogenic differentiation. Our findings demonstrate that serpin E2 overexpression indeed enhances these activities while altering the expression patterns of key regulatory factors, such as PDLIM5 and lamin A. These results are consistent with those obtained from protein interaction studies and provide further novel insights into our current understanding of this complex network.

Integrins are among the primary cellular components that regulate the mechanics of the ECM and its associated signalling proteins. They are connected to the cytoskeleton through focal adhesion proteins, such as vinculin and talin, enabling them to influence stem cells differentiation by transmitting mechanical signals from the ECM. This mechanism is crucial in many physiological processes because it enables stem cells to perceive and respond to changes in their microenvironment [37]. Although the directionality of these signal transduction pathways varies during physiological processes, they are closely interconnected. These pathways not only participate in fundamental life activities but also respond to various stimuli, such as mechanical stretch or alterations in chemical factors, providing support for the body to adapt to a constantly changing environment, such as cell proliferation, cell differentiation, cell survival, cell motility, cell apoptosis and embryogenesis [38, 39]. Specifically, research has demonstrated that the integrin αVβ3–actin axis regulates the early osteogenic differentiation of fibroblasts induced by cyclic stretch stimulation [40]. Our study provides further insights into this field. While previous studies have concentrated on specific aspects of ECM–cell interactions, our work offers a more comprehensive understanding of the underlying mechanisms involved. Specifically, we have demonstrated that the knockout of integrin β3 impairs the osteogenic differentiation of ASCs in mice, a finding that has not been previously reported. In our experiments, ASCs derived from integrin β3‐knockout mice exhibited impaired osteogenic differentiation compared to those from wild‐type mice (Figure 7B). This result suggests that this receptor may play an indispensable role in bone development and repair. Additionally, in these animal models, we found that the expression levels of PDLIM5 and serpin E2 in the femur of integrin‐β3‐knockout mice were lower than those in the femurs of wild‐type mice (Figure 7C–F). The overexpression of PDLIM5 and serpin E2 in mASCs lacking integrin β3 revealed their importance in mechanoregulation of osteogenic differentiation, further supporting our hypothesis regarding the significance of this pathway. Although both integrin αVβ5 and integrin αVβ3 can bind to several common ligands in the ECM, such as osteopontin, fibronectin and vitronectin [41], leading to functional similarities, previous studies have demonstrated that under mechanical stimulation, integrin αVβ3 is associated with the YAP signalling pathway [15], whereas integrin αVβ5 exhibits a closer relationship with the Wnt/β‐catenin pathway [42]. Additionally, existing research has shown that PDLIM5 promotes osteogenic differentiation through its interaction with the YAP pathway [13]. These results not only validate the findings of previous studies [43] but also pave the foundation for developing new future treatment strategies for related diseases, while facilitating a deeper understanding of the ECM–cell interactions. Similarly, this study emphasises the importance of exploring additional potential targets to improve regenerative medicine outcomes and expand clinical applications.

## 4 Conclusion

The mechanosensitive protein PDLIM5 serves as a crucial signal transduction molecule, exhibiting significantly increased expression during the osteogenic differentiation of hASCs subjected to chemical induction and mechanical stimulation. This upregulation is likely closely associated with the cells' adaptive responses to alterations in their external mechanical environment and is regulated by upstream proteins, such as serine protease E2 and integrin β3. PDLIM5 functions as an intracellular driving signal that facilitates the transmission of mechanical signals from the extracellular environment while activating the expression of osteogenic transcription factors. This process encompasses not only intracellular signalling pathways but also complex interactions between the ECM and cells. Collectively, PDLIM5 enhances the osteogenic differentiation of hASCs through the integrin β3/serpin E2‐PDLIM5/lamin A signalling axis. Furthermore, elucidating the interactions among various receptors can facilitate the development of more targeted drug designs aimed at achieving precision medicine objectives and improving patient quality of life.

## 5 Experimental Section

### 5.1 Cell Culture and Osteogenic Differentiation

Human ASCs were purchased from Cyagen Biosciences (HUXMD‐90011, Suzhou, China). We adhere to the stem cell standards published by Cell Proliferation and the International Society for Stem Cell Research (ISSCR) for the quality control of mesenchymal stromal cells (MSCs), including experimental protocols, instructions for use, storage guidelines and waste disposal requirements [44]. Adherent hASCs were cultured in a growth medium (GM) containing high‐sugar Dulbecco's modified Eagle's medium (H‐DMEM; Gibco, Waltham, MA), 10% fetal bovine serum (FBS; Gibco), 1% penicillin/streptomycin (Gibco) and 0.2% plasmocin prophylactic (Invivo Gen, San Diego, CA) in a humidified incubator maintained at 37°C and 5% CO_2_. The human ASCs at passages 3–7 were used for subsequent experiments. Murine adipose stem cells (mASCs) were isolated from the inguinal fat pads of 4‐week‐old C57BL/6 mice, which were minced and digested with 0.1%–0.15% (w/v) collagenase type I (Sigma‐Aldrich, USA) at 37°C for 40–60 min. Afterwards, the digestion was terminated with an equal volume of serum‐containing DMEM.

The extract was centrifuged at 1200 rpm for 10 min. The supernatant was then discarded, and the mASCs were suspended in GM, seeded in a petri dish, and cultured at 37°C and 5% CO_2_ in a humidified incubator for 3–4 days. The growth medium was changed every 2 days. When the confluency reached 80%, the cells were passaged. The purity of hASCs/mASCs was guaranteed by the third passage. When the purified hASCs or mASCs reached 80% confluence, the GM was replaced with the osteogenic induction medium (OS) containing DMEM medium, 5% FBS, 1% penicillin/streptomycin, 100 nM dexamethasone (Sigma‐Aldrich), 37.5 mg/L ascorbic acid (Sigma‐Aldrich), 10 mM β‐glycerophosphate (Sigma‐Aldrich) and 10 nM VitD3 (Solarbio, Beijing, China). The cells were then grown for 7 or 21 days, and the medium was changed every 2 days.

### 5.2 Preparation of Single‐Cell Suspension

Single‐cell suspensions of the hASCs (third passage) belonging to the hASC‐GM and hASC‐OS groups were prepared as previously described [24]. Briefly, the cells from the hASC‐GM and hASC‐OS groups were digested with 0.25% trypsin–EDTA when they reached a confluency of 70%–80% (Thermo Fisher Scientific, Waltham, MA). The cells were then centrifuged at 1000 rpm for 5 min. After removing the supernatants, the cell pellets were resuspended in PBS containing 0.04% BSA. The cell concentration was determined using the Aber Countstar (IC1000, Aber Instruments Ltd., Ceredigion, UK). The cells were diluted to obtain a final concentration of 0.8–1.2 × 10^6^ cells/mL. Cell viability was determined using trypan blue (Sigma). Samples with more than 80% live cells and 90% single cells were used for subsequent experiments.

### 5.3 Single‐Cell RNA‐Seq Library Preparation and Sequencing

Single‐cell RNA sequencing (scRNA‐seq) was performed using the 10× Genomics Chromium Platform, and the scRNA‐seq libraries were prepared using the Chromium Single Cell 30 library kit (V2, Pleasanton, CA) according to the manufacturer's protocol. After library construction and cDNA amplification, the concentration of cDNA was determined using Qubit (Thermo Fisher Scientific) and the fragment length of the libraries was determined using the Agilent 2100 Bio‐analyser (Agilent Technologies, California). Finally, the barcoded libraries were sequenced using the HiSeq 2000 sequencer (Illumina, California).

### 5.4 ScRNA‐Seq Data Processing

Basecalling, adaptor trimming and de‐multiplexing of the raw sequencing data were performed using the Cell Ranger v. 3.0 software package. The quality control, clustering and differentiation analyses were performed using the R package Seurat v. 4.0 as previously described [45]. Briefly, cells with the following criteria were excluded: unique genes < 100 or > 8000, mitochondrial counts > 7.5% or < 0.5% and number of unique molecular identifiers (UMI) < 1000 or > 100,000. Principal component analysis (PCA) and uniform manifold approximation and projection (UMAP) were used to perform dimensionality reductions of the top 40 principal components (PCs). The cells from each sample were clustered using the graph‐based clustering approach, and the ‘resolution’ parameter was set to 0.4.

### 5.5 Alkaline Phosphatase Staining

The hASCs and mASCs were seeded in 6‐well plates and special collagen‐coated silica gel membrane plates at a density of 1 × 10^5^ cells per well. The cells were chemically induced in the 6‐well plates, and alkaline phosphatase staining was performed on days 0, 7, 14 and 21 after induction. The alkaline phosphatase staining of cells induced by stretching on the silica gel plates was performed immediately after stretching on days 0, 1, 4 and 7.

Briefly, the cells were washed three times with PBS (Boster, Wuhan, China), fixed with 4% paraformaldehyde for 15 min, and further washed three times with PBS. The cells were then incubated for 40 min with the BCIP/NBT working solution (Beyotime, Shanghai, China), which was prepared according to the manufacturer's instructions. The stained cells were observed and photographed under a microscope (Olympus, Tokyo, Japan).

### 5.6 Protein Extraction

Protein extracts were prepared from cells stimulated with the osteogenic induction medium on days 0, 1, 4, 7, 14 and 21, and from cells induced by mechanical strain on days 0, 1, 4 and 7. Briefly, the cells were washed three times with pre‐cooled PBS. Afterwards, the cells were incubated with the cell lysis buffer from the whole protein extraction kit (Key GEN BioTECH, Nanjing, China). The adherent cells were scraped with a cell scraper. The cells were then gently agitated at 4°C on a refrigerated shaker for 30 min. The cell lysate was then centrifuged at 12,000 *g* at 4°C for 10 min. The supernatant was harvested, mixed with 5X sample loading buffer and boiled in a 100°C water bath for 5–10 min.

### 5.7 Western Blotting

Equal amounts of protein samples were separated on a 10% SDS polyacrylamide gel and transferred onto a 0.22‐μm polyvinylidene fluoride (PVDF) membrane (Millipore, Waltham, MA). The PVDF membrane was blocked with 5% skim milk for 1 h at room temperature. The blots were then incubated overnight at 4°C with the following primary antibodies: anti‐Serpin E2 antibody (ab154591, Abcam, Cambridge, UK, dilution 1:1500), anti‐Integrin β3 antibody (orb214124, biorbyt, dilution 1:1000), anti‐PDLIM5 antibody (ab85967, dilution 1:1500; 10530‐1‐AP, proteintech, USA, dilution 1:1000), anti‐α‐actinin 1 antibody (ab68194, dilution 1:1500), anti‐lamin A antibody (ab8984, dilution 1:1500; 10298‐1‐AP, proteintech, USA, dilution 1:1000), anti‐osteopontin (OPN) antibody (ab69498, dilution 1:1500), anti‐RUNT‐related transcription factor 2 (RUNX2) antibody (CST#12556S, Cell Signalling Technology, Danvers, MA, dilution 1:1000) and anti‐GAPDH antibody (AP0063, dilution 1:5000, Bioworld, Bloomington, MN). The blots were washed with TBST three times for 15 min each and then incubated with the secondary antibodies (Goat Anti‐Rabbit and Goat Anti‐Mouse, Fudebio, Hangzhou, China) at room temperature for 1 h (20°C–25°C). Subsequently, after washing the blots three times with TBST for 15 min each, the protein bands were developed and visualised using enhanced chemiluminescence (ECL) (Fudebio) chromogenic substrate. Signal intensity was assessed using a Tanon‐5500 chemiluminescence detection system (Tanon Science & Technology Ltd., Shanghai, China). The immunoreactive bands were quantitatively analysed using Image J software (National Institutes of Health, Bethesda, MD).

### 5.8 Immunofluorescence

The cells were seeded on coverslips in 24‐well plates, washed three times with PBS for 2 min each and fixed with 4% paraformaldehyde for 15 min. After washing three times with PBS, they were permeabilised with 0.3% Triton X‐1000 for 5 min. After washing three times again with PBS, the cells were incubated with 2% bovine serum albumin (BSA) at room temperature (20°C–25°C) for 1 h. Subsequently, the cells were incubated overnight at 4°C with the following primary antibodies: anti‐Serpin E2 antibody (sc‐365650, Santa Cruz Biotechnology, USA, dilution 1:100), anti‐Integrin β3 antibody (MA5‐32077, Thermo Fisher Scientific, dilution 1:500), anti‐PDLIM5 antibody (h00010611‐M01, Abnova Company, Taipei, Taiwan, dilution 1:1000), anti‐α‐actinin1 antibody (ab68194, dilution 1:1000), anti‐Lamin A antibody (ab8984, dilution 1:1000) and anti‐RUNX2 antibody (CST#12556S, dilution 1:1000). The cells were washed three times with PBS for 10 min each and then incubated with the corresponding secondary antibody (Alexa Fluor Plus 488 and 568, Invitrogen, Waltham, MA) in the dark at room temperature (20°C–25°C) for 1 h. Afterwards, the cells were washed three times with PBS for 10 min each time. The cells on the coverslips were mounted on the glass slides using the 2‐Phenylindole (DAPI) mounting medium. The stained cells were observed and photographed using a confocal microscope (Carl Zeiss, LSM 880, Jena, Germany).

### 5.9 Lentivirus Transfection

To knock down PDLIM5 and overexpress PDLIM5 and serpin E2 expression, lentiviruses for PDLIM5‐knockdown and PDLIM5‐overexpression and serpin E2‐overexpression and all types of lentiviruses with their corresponding controls were generated by GeneChem (GeneChem Co. Ltd., Shanghai, China). A pre‐experiment lentiviral infection was first performed to optimise the best conditions for infection, including MOI and infection time. Subsequently, the cells were transfected with the corresponding lentiviruses for 12 h. The medium containing the virus was then replaced with GM, and the cells were further cultured. Subsequent changes in the medium were in accordance with the cell growth status. After 72–96 h of culturing, the cell transfection efficiency was observed under a fluorescence microscope.

### 5.10 Cell Proliferation Assay (CCK8 Assay)

The cells were seeded in a 96‐well plate at a density of 5 × 10^3^ cells per well. The cells were washed three times with PBS after adherence. The CCK8 reaction solution (Dojindo, Kumamoto, Japan) was prepared according to the manufacturer's instructions, added to the wells and incubated for 1 h. The absorbance was measured at 450 nm using a microplate reader (Thermo Fisher, Thermo Scientific Multiskan FC, Waltham, MA).

### 5.11 Wound Healing

The cells were seeded in 12‐well plates at a density of 5 × 10^4^ cells per well and divided into four groups: NC1, OE‐Serpin E2, NC2, sh‐PDLIM5. After the cells were adherent, the monolayer of cells was scratched with a 200‐μL pipette tip. The exfoliated cells were removed by washing three times with PBS. The cells were then incubated with a serum‐free medium for 0, 8, 12, 24 and 48 h. The migration ability of the cells was observed under a bright‐field microscope (Olympus, Tokyo, Japan).

### 5.12 Transwell Migration Assay

The transwell chamber (Corning, NY) was placed in a 24‐well plate. Afterwards, 300 μL of the cell suspension (2 × 10^5^ cells/mL of serum‐free medium) was added to the upper chamber, and 700 μL of GM was added to the lower chamber. After incubation at 37°C for 12–16 h, the chamber was removed. The upper and lower chambers were gently washed with PBS, fixed with 4% formaldehyde for 15 min and washed with PBS three times. The cells were stained with 1% crystal violet staining solution (Solarbio Company, Beijing, China) for 1 h. The excess staining solution was washed with PBS. The non‐migrated cells were wiped off with a cotton swab, and the migrated cells were observed and quantified using a light microscope (Olympus, Tokyo, Japan).

### 5.13 Cyclic Strain Loading

The specialised collagen‐coated silicone membrane plates (Bioflex, Flexcell International, NC) were seeded with 1 × 10^5^ cells per well in GM. After 48 h, cyclic tensile stress with an intensity of 10% and a frequency of 0.5 HZ was applied using the Flexcell FX‐5000 system (Flexcell International). The silicone plate was then stretched by tensile stress for 2 h/day, and the medium was changed every 2 days.

### 5.14 Radiographic Analysis

The posterior limbs of the mice (Itg β3^−/−^ and WT) were obtained and preserved in 3.7% PFA for 48 h at room temperature (20°C–25°C). The distal femur was scanned for bone formation using a micro‐computed tomography (micro‐CT) imaging system (ZKKS‐MCT‐Sharp, Zhongke Kaisheng Medical Technology, Guangzhou, China) with the following scan parameters: the scanning voltage was set to 70 kV, power to 7 W, stacking of four frames, angle gain 0.72° with an exposure time of 100 ms and a rotation of one circle to complete the scanning. ZKKS‐Scan software was used to reconstruct the scanned images and examine the trabecular bone characteristics (ZKKS‐MicroCT 4.1). Bone mineral density (BMD) and bone volume‐to‐tissue volume ratio (BV/TV) were also determined.

### 5.15 Haematoxylin and Eosin (H&E) Staining

The femurs and tibias (including the knee joints) of the wild‐type (WT) and integrin β3 flox mice (aged 6–8 weeks) were fixed, decalcified, paraffin‐embedded and sectioned. The sections were then dewaxed with Xylene and rehydrated with gradient alcohol solutions. The sections were stained with H&E (Solarbio) solutions. The sections on slides were then mounted and sealed with neutral gum.

### 5.16 Immunohistochemical and Immunofluorescence Staining

Regarding immunohistochemical staining, the sections were incubated with the sodium citrate buffer (Boster) for antigen repair. The sections were then washed with pure water and incubated with endogenous peroxidase blockers for 20 min. Afterwards, the sections were incubated overnight at 4°C with the corresponding primary antibodies. After washing thrice with PBS for 5 min each, they were incubated with the secondary antibody (Goat Anti‐Mouse and Rabbit HRP, ORIGENE, Rockville, MD) at 37°C for 1 h. The sections were washed with PBS, counterstained with haematoxylin and sealed with neutral gum. The positive staining areas of the two groups were observed using an optical microscope (Olympus, Tokyo, Japan).

For tissue immunofluorescence staining, the formalin‐fixed paraffin‐embedded tissue sections were dewaxed and rehydrated before heat‐induced antigen retrieval was performed at a pH of 9.5 (i.e., 0.1 M Tris/HCL + 5% Urea, pH 9.5). The tissue sections were pre‐rinsed in PBS for 30 min and then incubated with the primary antibody at 4°C overnight. The tissue sections were washed twice for 30 min in TBST, then incubated with the blocking buffer for 30 min. Afterwards, the tissue sections were incubated with a fluorophore‐conjugated secondary antibody for 90 min in the blocking buffer and then washed once for 30 min. The tissue section was incubated with Hoechst stain for 30 min and then washed in the buffer. The tissue section was also washed once for 30 min. Coverslips were placed over the tissue sections with the appropriate water‐based mounting media.

### 5.17 Statistical Analysis

Statistical analysis was performed using GraphPad Prism software (version 8.0). Statistical data were expressed as the mean ± standard deviation of at least three independent experiments. The differences between groups were analysed using the Student's *t*‐test and one‐way ANOVA. *p*‐values < 0.05 were considered statistically significant.

## Author Contributions

J.O., J.D., K.H. and Y.Y. conceived and designed the experiments. Y.Y., S.W., Y.W., J.T., J.L., J.W., W.L. and J.Z. performed the experiments. Y.Y., S.W., Y.L., J.O. and J.D. analysed the data and wrote the manuscript. Y.Y., S.W., A.U.K., M.A.K., J.O., J.D. and K.H. revised the manuscript. All the authors read and approved the final manuscript. All the authors agree to take responsibility for the integrity and accuracy of the data presented in this article.

## Ethics Statement

This animal experiment was approved by the Institutional Animal Care and Use Committee (IACUC) of the Southern Medical University (Resolution NO.: L2018147, Date of Resolution: 09/10/2018).

## Consent

The authors agree with the publication of all the data involved in this article. No data from other entities was used in this study.

## Conflicts of Interest

The authors declare no conflicts of interest.

## Supporting information


**FIGURE S1.** (A) GO annotation results. (B) Significantly enriched GO term. C. Significantly enriched KEGG pathway.

**FIGURE S2.** (A) CCK8 assay results demonstrate the proliferation status of the NC1 group and shPDLIM5 group in hASCs. (B) Wound healing assay results reveal the motility status of the NC1 group and shPDLIM5 group in hASCs. (C) Transwell migration assay results indicate the migration ability of the NC1 group and shPDLIM5 group in hASCs. As shown, PDLIM5 knockdown significantly reduced the proliferation and migration capabilities of the hASCs. Scale bar = 200 μm. NC1: empty plasmid negative control group; sh‐PDLIM5: PDLIM5 knockdown experimental group.

**FIGURE S3.** Distribution map of the top 2 tag genes in different subgroups. The intensity of gene expression in the samples is represented by the colour depth.

**FIGURE S4.** Expression distribution map of the top 2 differentially expressed genes in each subgroup.

**FIGURE S5.** Violin diagram of the top 2 differentially expressed genes in each subgroup.

**FIGURE S6.** (A) CCK8 assay results demonstrate the proliferation status of the NC2 group and OE‐Serpin E2 group in hASCs. (B) Wound healing assay results reveal the motility status of the NC2 group and OE‐Serpin E2 group in hASCs. (C) Transwell migration assay results indicate the migration ability of the NC2 group and OE‐Serpin E2 group in hASCs. As shown, upregulation of serpin E2 increased the proliferation and migration capabilities of the hASCs. Scale bar = 200 μm. NC2: empty plasmid negative control group; OE‐Serpin E2: Serpin E2 overexpression experimental group.

## Data Availability

The data that support the findings of this study are available from the corresponding author upon reasonable request.
