# Supplementary figures and images for: Mechanotransduction Mediated by PDLIM5: The Critical Role of Serpin E2/Integrin β3‐Cytoskeleton‐Nucleoskeleton Axis in Mechanical Osteogenic Programming
Source: Cell Prolif. 2025 Jun 3;58(12):e70067. doi: 10.1111/cpr.70067 (PMC12686131; doi:10.1111/cpr.70067)

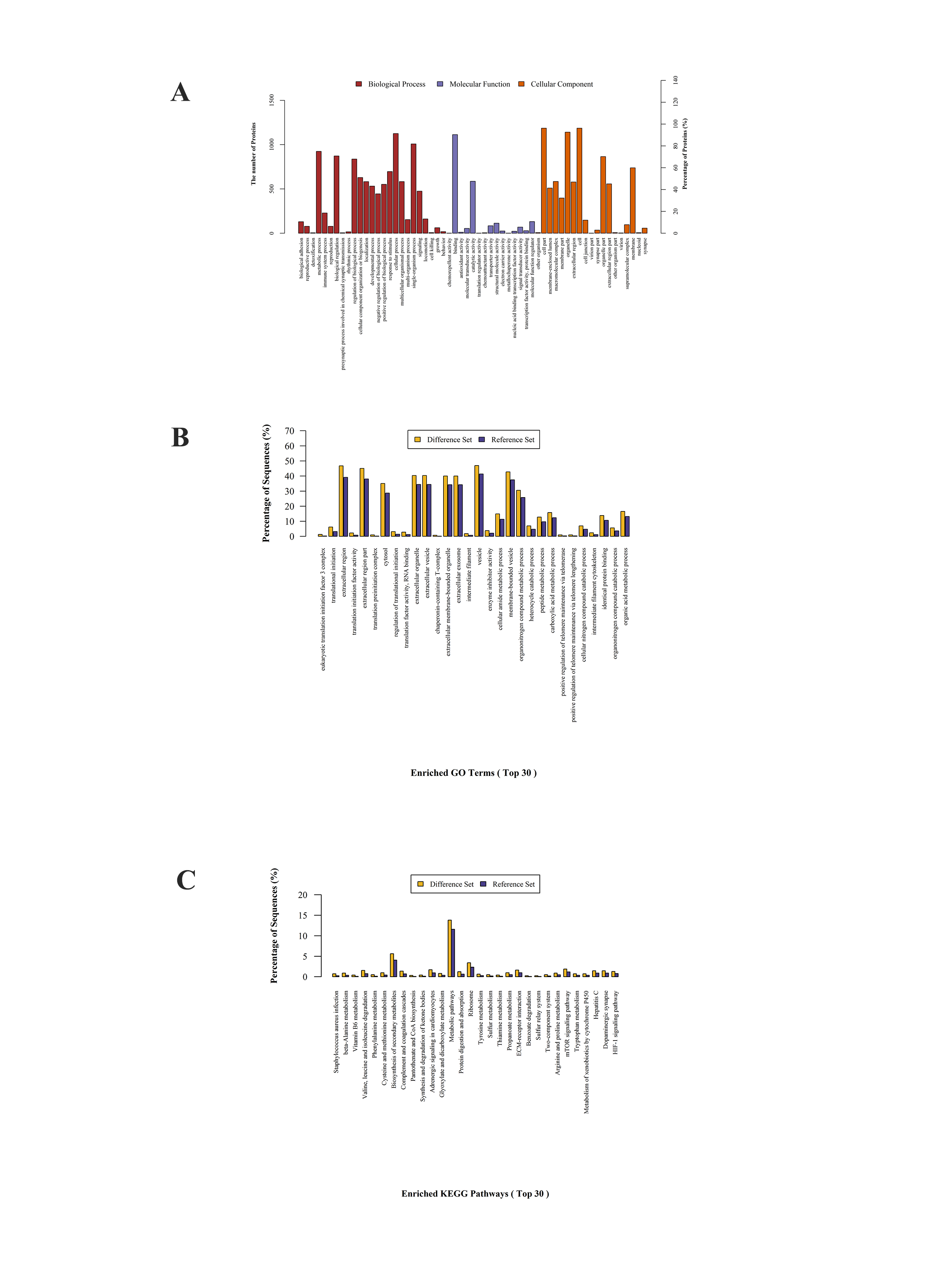

Supplement: Supplementary file 1 — FIGURE S1. (A) GO annotation results. (B) Significantly enriched GO term. C. Significantly enriched KEGG pathway. [file CPR-58-e70067-s005.tif]

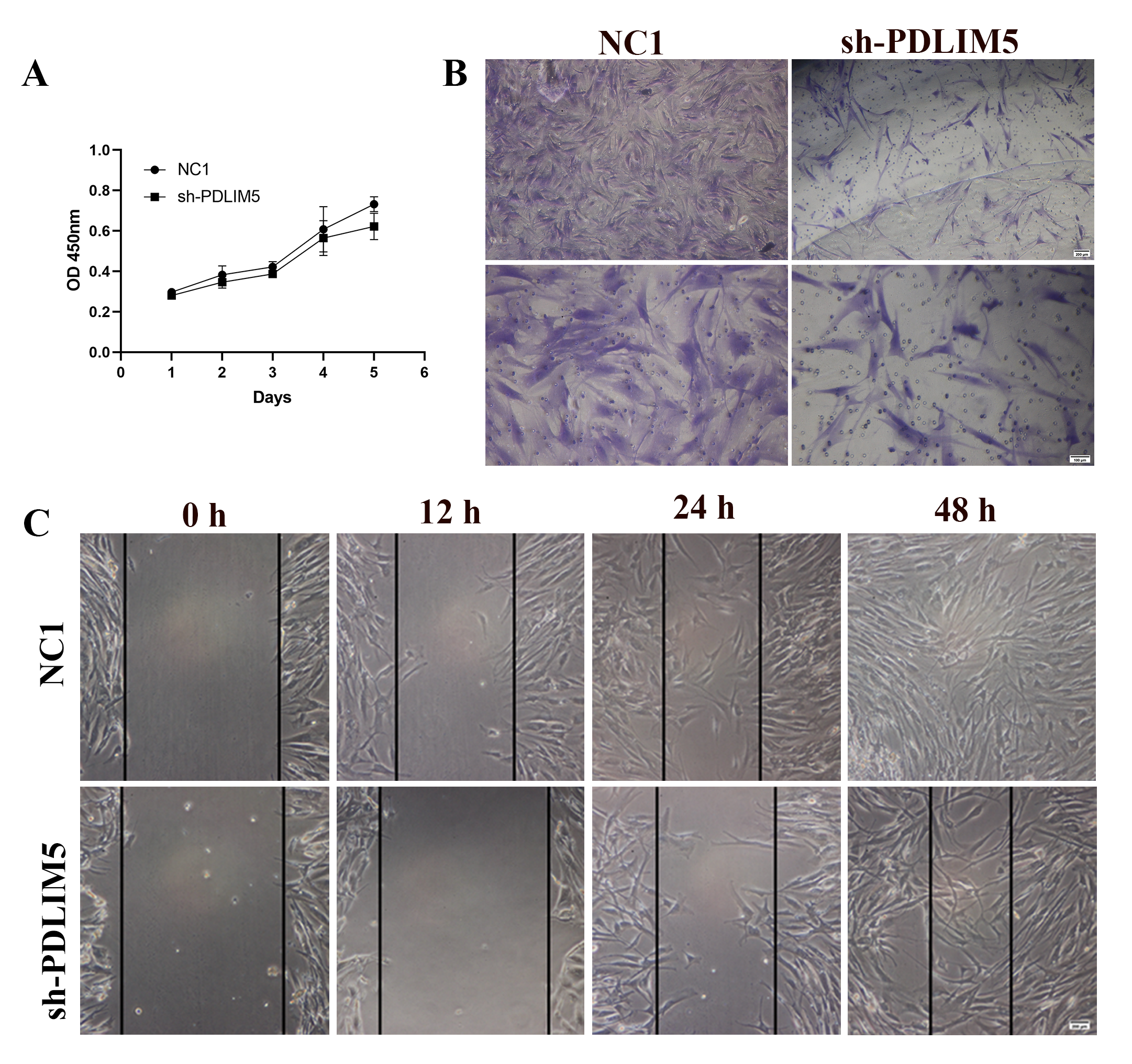

Supplement: Supplementary file 2 — FIGURE S2. (A) CCK8 assay results demonstrate the proliferation status of the NC1 group and shPDLIM5 group in hASCs. (B) Wound healing assay results reveal the motility status of the NC1 group and shPDLIM5 group in hASCs. (C) Transwell migration assay results indicate the migration ability of the NC1 group and shPDLIM5 group in hASCs. As shown, PDLIM5 knockdown significantly reduced the proliferation and migration capabilities of the hASCs. Scale bar = 200 μm. NC1: empty plasmid negative control group; sh‐PDLIM5: PDLIM5 knockdown experimental group. [file CPR-58-e70067-s004.tif]

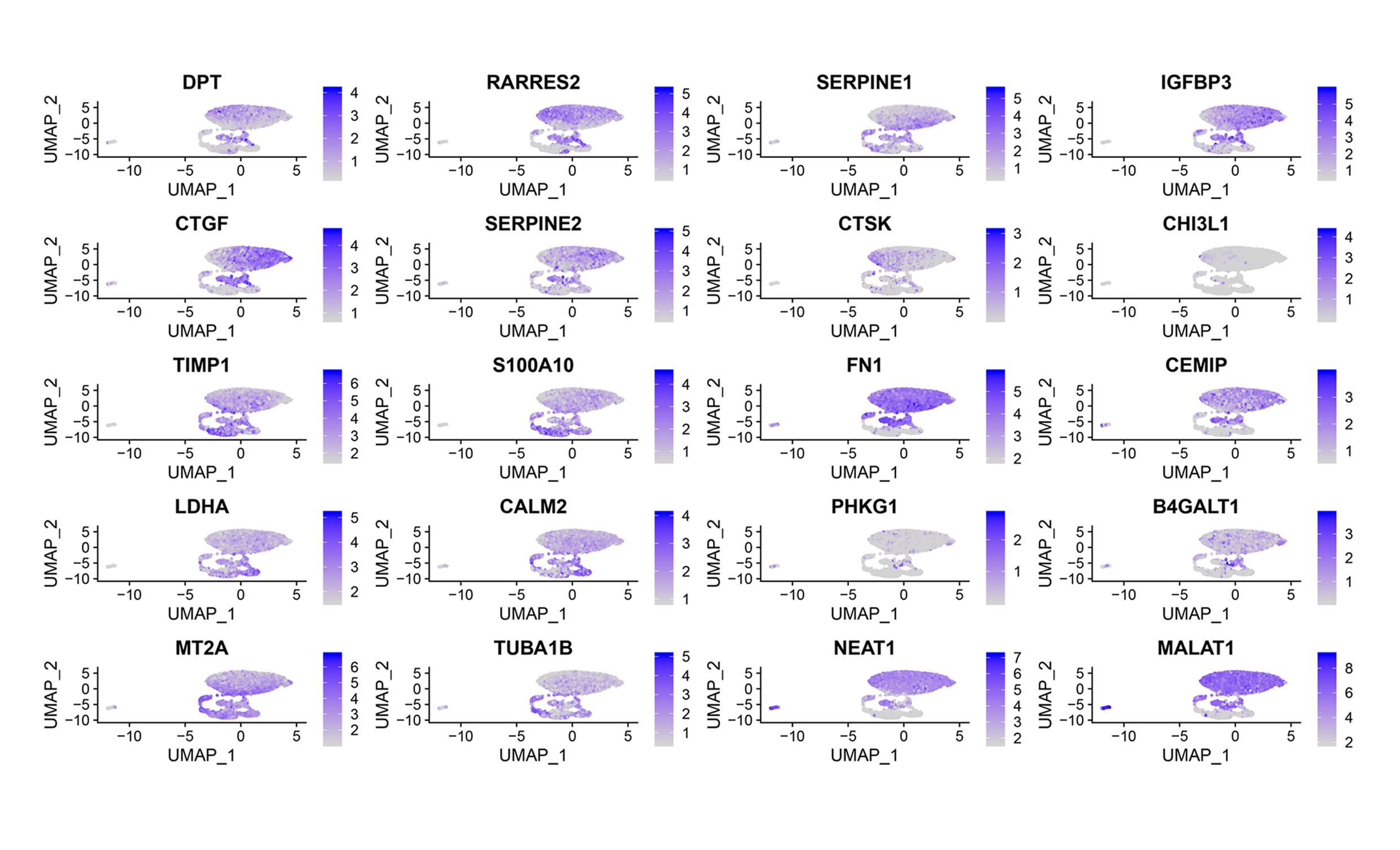

Supplement: Supplementary file 3 — FIGURE S3. Distribution map of the top 2 tag genes in different subgroups. The intensity of gene expression in the samples is represented by the colour depth. [file CPR-58-e70067-s002.tif]

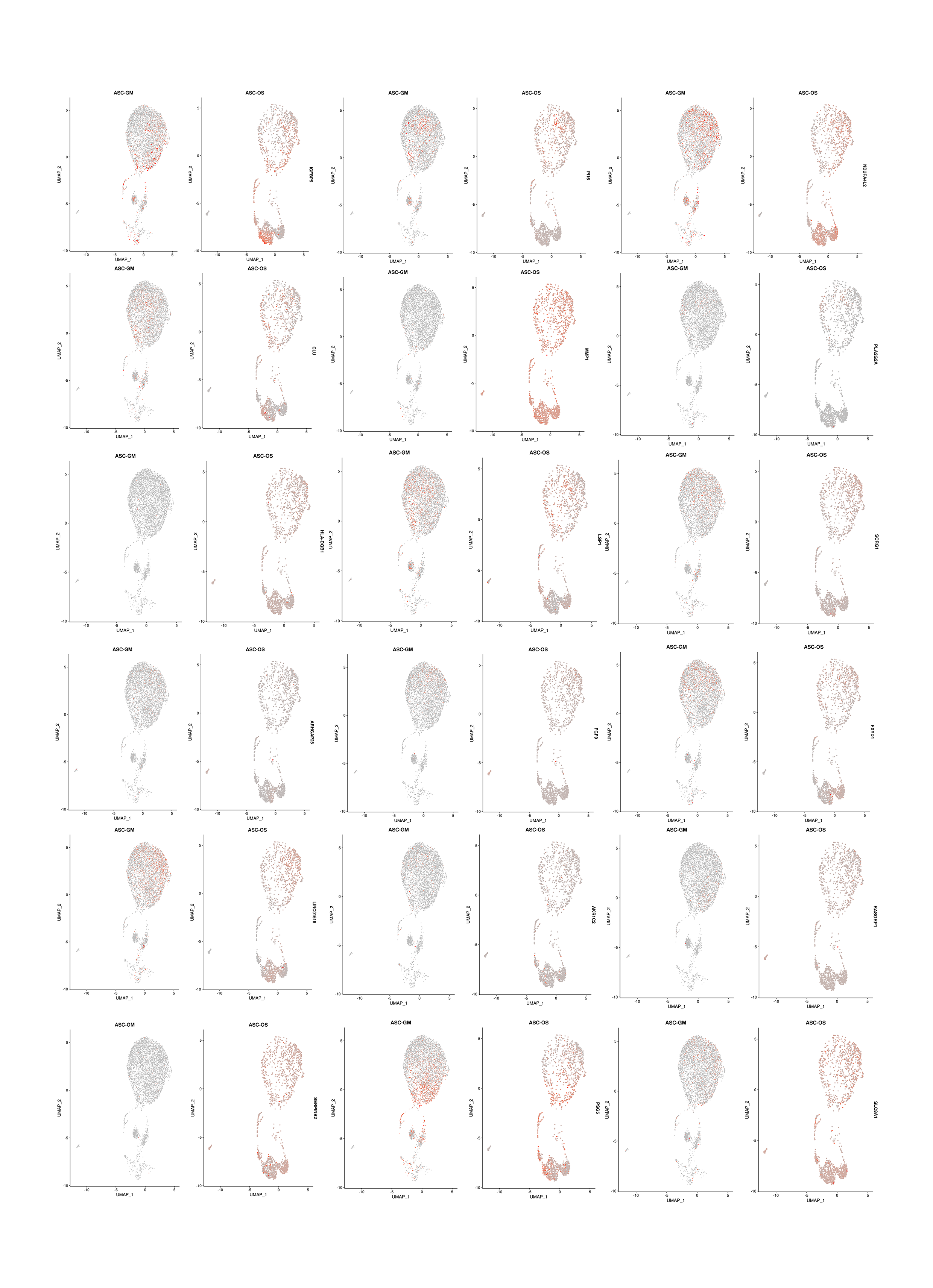

Supplement: Supplementary file 4 — FIGURE S4. Expression distribution map of the top 2 differentially expressed genes in each subgroup. [file CPR-58-e70067-s003.tif]

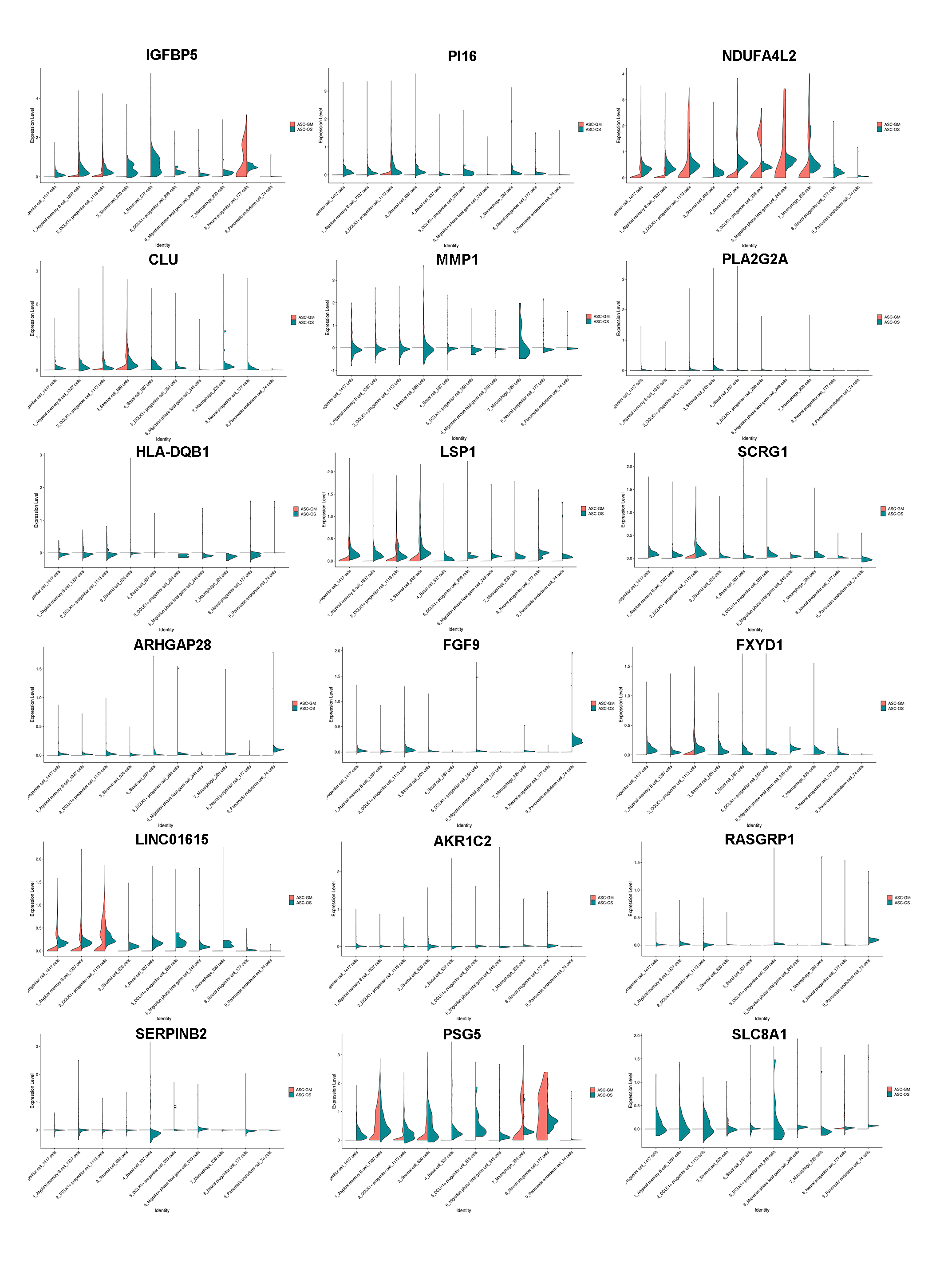

Supplement: Supplementary file 5 — FIGURE S5. Violin diagram of the top 2 differentially expressed genes in each subgroup. [file CPR-58-e70067-s006.tif]

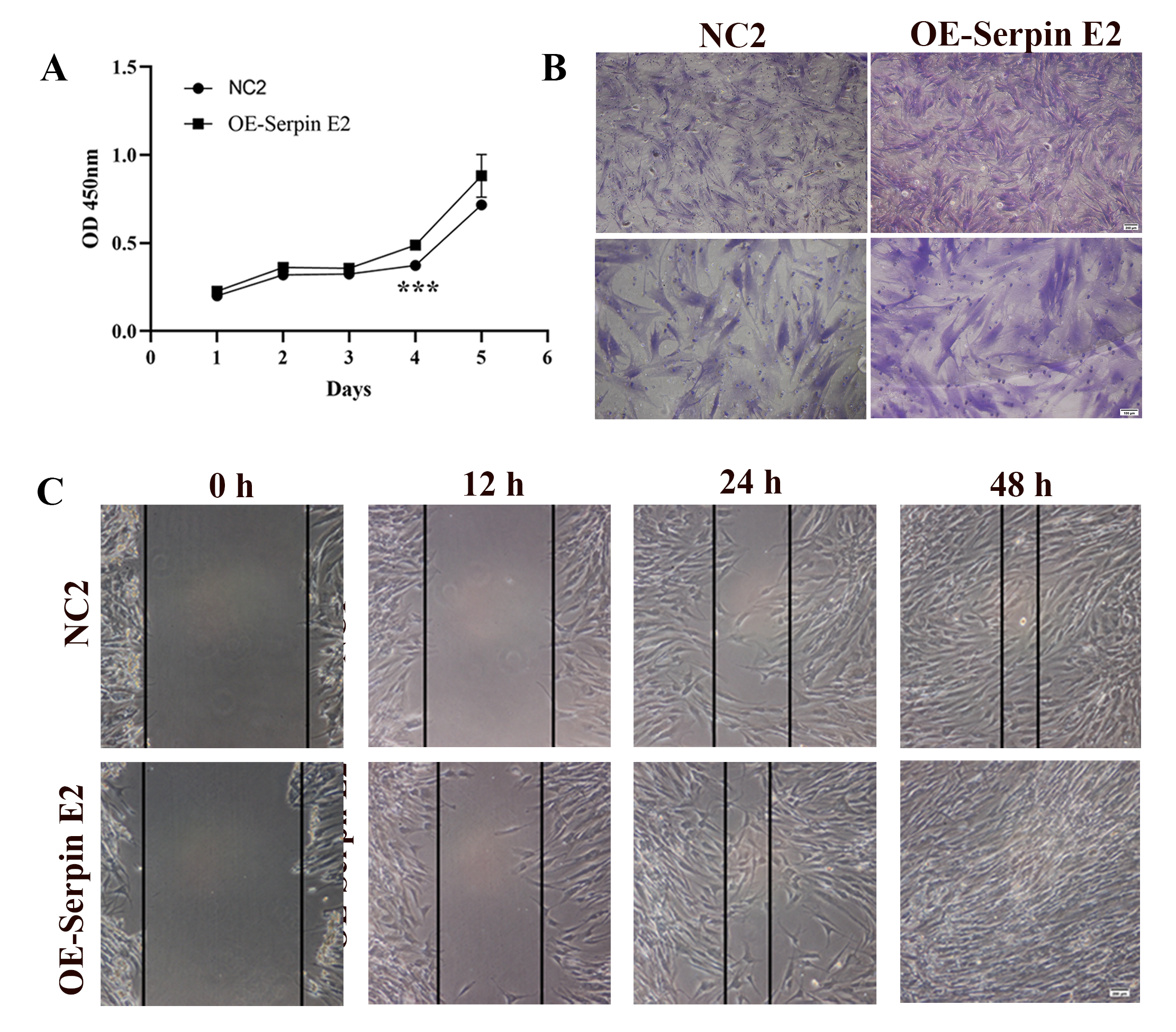

Supplement: Supplementary file 6 — FIGURE S6. (A) CCK8 assay results demonstrate the proliferation status of the NC2 group and OE‐Serpin E2 group in hASCs. (B) Wound healing assay results reveal the motility status of the NC2 group and OE‐Serpin E2 group in hASCs. (C) Transwell migration assay results indicate the migration ability of the NC2 group and OE‐Serpin E2 group in hASCs. As shown, upregulation of serpin E2 increased the proliferation and migration capabilities of the hASCs. Scale bar = 200 μm. NC2: empty plasmid negative control group; OE‐Serpin E2: Serpin E2 overexpression experimental group. [file CPR-58-e70067-s001.tif]
